# Integrative Analysis of Hereditary Nonpolyposis Colorectal Cancer: the Contribution of Allele-Specific Expression and Other Assays to Diagnostic Algorithms

**DOI:** 10.1371/journal.pone.0081194

**Published:** 2013-11-20

**Authors:** Laura De Lellis, Gitana Maria Aceto, Maria Cristina Curia, Teresa Catalano, Sandra Mammarella, Serena Veschi, Fabiana Fantini, Pasquale Battista, Vittoria Stigliano, Luca Messerini, Cristina Mareni, Paola Sala, Lucio Bertario, Paolo Radice, Alessandro Cama

**Affiliations:** 1 Department of Pharmacy, “G. d’Annunzio” University, Chieti, Italy; 2 Department of Experimental and Clinical Sciences, “G. d’Annunzio” University, Chieti, Italy; 3 Department of Medical, Oral and Biotechnological Sciences, “G. d’Annunzio” University, Chieti, Italy; 4 Unit of Molecular Pathology and Genomics, Aging Research Center, “G. d’Annunzio” University Foundation, Chieti, Italy; 5 Department of Clinical and Experimental Medicine, University of Messina, Messina, Italy; 6 Department of Gastroenterology, Unit of Hereditary Colorectal Cancer, National Cancer Institute, Regina Elena (IRE), Rome, Italy; 7 Section of Pathological Anatomy, Department of Medical and Surgical Critical Care, University of Florence, Florence, Italy; 8 Department of Internal Medicine, University of Genova, Genova, Italy; 9 Unit of Hereditary Digestive Tract Tumors, Department of Preventive and Predictive Medicine, Fondazione IRCCS Istituto Nazionale dei Tumori, Milan, Italy; 10 Unit of Molecular bases of genetic risk and genetic testing, Department of Preventive and Predictive Medicine, Fondazione IRCCS Istituto Nazionale dei Tumori, Milan, Italy; Ohio State University Medical Center, United States of America

## Abstract

The identification of germline variants predisposing to hereditary nonpolyposis colorectal cancer (HNPCC) is crucial for clinical management of carriers, but several probands remain negative for such variants or bear variants of uncertain significance (VUS). Here we describe the results of integrative molecular analyses in 132 HNPCC patients providing evidences for improved genetic testing of HNPCC with traditional or next generation methods. Patients were screened for: germline allele-specific expression (ASE), nucleotide variants, rearrangements and promoter methylation of mismatch repair (MMR) genes; germline *EPCAM* rearrangements; tumor microsatellite instability (MSI) and immunohistochemical (IHC) MMR protein expression. Probands negative for pathogenic variants of MMR genes were screened for germline *APC* and *MUTYH* sequence variants. Most germline defects identified were sequence variants and rearrangements of MMR genes. Remarkably, altered germline ASE of MMR genes was detected in 8/22 (36.5%) probands analyzed, including 3 cases negative at other screenings. Moreover, ASE provided evidence for the pathogenic role and guided the characterization of a VUS shared by 2 additional probands. No germline MMR gene promoter methylation was observed and only one *EPCAM* rearrangement was detected. In several cases, tumor IHC and MSI diverged from germline screening results. Notably, *APC* or biallelic *MUTYH* germline defects were identified in 2/19 probands negative for pathogenic variants of MMR genes. Our results show that ASE complements gDNA-based analyses in the identification of MMR defects and in the characterization of VUS affecting gene expression, increasing the number of germline alterations detected. An appreciable fraction of probands negative for MMR gene variants harbors *APC* or *MUTYH* variants. These results indicate that germline ASE analysis and screening for *APC* and *MUTYH* defects should be included in HNPCC diagnostic algorithms.

## Introduction

Hereditary nonpolyposis colorectal cancer (HNPCC), also known as Lynch syndrome, is the most frequent form of autosomal dominant predisposition to colorectal cancer (CRC) [1]. Genetic diagnosis of germline defect carriers in affected families is fundamental for an efficient clinical surveillance and allows targeted chemoprevention that was recently shown to substantially reduce cancer incidence in these patients [2]. However, the identification of pathogenic germline defects in this syndrome is not trivial, as reflected by the wide fluctuation in the rate of genetic alterations identified in different studies and by the relevant number of variants with uncertain significance (VUS) detected [3-6]. The variable rate of alterations detected reflects in part differences in sensitivity of the molecular methods utilized and in part the fact that clinical diagnosis does not completely account for the underlying genetic heterogeneity, even when the selection of probands is based on strict Amsterdam criteria I (AC-I) or Amsterdam criteria II (AC-II) [7]. The majority of HNPCC patients are linked to germline mismatch repair (MMR) defects and develop tumors with high levels of microsatellite instability (MSI-H), the hallmark of MMR deficiency. Germline *MLH1* or *MSH2* variants are identified in most of these patients, whereas variants in other MMR genes are detected in a smaller fraction of cases [1-6]. Notably, also germline deletions affecting the 3’ end of the epithelial cell adhesion molecule gene (*EPCAM*) may cause HNPCC through hypermethylation and silencing of the downstream *MSH2* promoter in EPCAM-expressing tissues [8]. *EPCAM* deletions were reported at a relatively high frequency (16-21%) in different studies conducted in cases negative for germline MMR defects [9,10]. The highest frequency (up to 33%) of *EPCAM* rearrangements was observed in the subset of MSI-H patients negative for germline MMR alterations and lacking MSH2 tumor expression [11,12], but the overall frequency of these rearrangements in series of HNPCC probands, unselected for mutational status or MSH2 tumor immunostaining, has not been evaluated. 

In addition to MMR genes and *EPCAM*, other genes play a role in this genetically heterogeneous syndrome. A relatively high proportion of families meeting AC has “familial colorectal cancer type X” (FCCTX), a colorectal cancer aggregation with no evidence of germline or tumor-associated MMR defects [4]. For the majority of these families, the genetic alteration responsible for colon cancer predisposition is not known, although defects in non-MMR genes, such as *MUTYH*, *OGG1* or *BMPR1A*, are occasionally detected [13-15]. In this regard, also *APC* variants associated to very attenuated phenotypes may overlap with HNPCC [16], further widening the range of CRC predisposing genes to be screened.

Based on the above considerations, traditional genetic testing in HNPCC focused on the analysis of MMR genes and several diagnostic algorithms were proposed to optimize this screening [17-21]. However, these algorithms may limit the sensitivity of genetic testing, underestimating carriers of pathogenic variants in MMR genes. Recently, a highly processive gDNA assay (ColoSeq, University of Washington, Seattle, WA) based on targeted capture and next-generation sequencing (NGS) was designed to simultaneously analyze MMR-related and -unrelated genes in HNPCC [22]. NGS-based testing can overcome some of the limitations of low-throughput methods, but even this approach has some drawbacks. For instance, NGS does not provide insights into the pathogenic role of VUS that are more likely to be detected by these highly processive methods. Moreover, genomic-based approaches are not designed to define the pathogenic potential of *cis*- and trans-acting variants that affect gene expression. These limitations might be in part overcome by cDNA-based assays, such as the analysis of allele-specific expression (ASE) that has the potential to uncover germline defects predisposing to colorectal cancer even when a pathogenic variant has not been ascertained or the role of the variants detected is unclear [23-27].

In the present study, we illustrate the results of integrative analyses conducted on a series of 132 Italian HNPCC patients using previously developed and novel assays for the screening of germline and tumor defects. We show that germline ASE analysis complements gDNA-based assays in the identification and characterization of defects predisposing to CRC. Our integrative approach also shows that inclusion of *MUTYH* and *APC* in the screening increases the number of pathogenic variants detected. Considering the number of patients analyzed, the panel of genes screened and the range of methods employed, this study provides indications for clinical translation of HNPCC genetic testing that may be applied to traditional or NGS approaches. In particular, our results support the inclusion of ASE analysis and screening of polyposis genes in algorithms for genetic diagnosis of HNPCC.

## Materials and Methods

### Patients and integrative screening strategy

We studied 132 unrelated AC-I or AC-II patients previously recruited at different Italian institutions. DNA and RNA were extracted as previously described [25]. All study participants gave written informed consent after verbal counseling and the study was approved by the Ethics Committee of the University of Chieti. Tumor MSI and IHC analyses were conducted in probands with available tumor samples. All patients, irrespective of the results of tumor MSI and IHC analyses, underwent screening for germline nucleotide substitutions in MMR genes as detailed below. Probands negative for pathogenic nucleotide substitutions were further tested for extended germline rearrangements in *MSH2*, *MLH1* and *EPCAM*, followed by screening for germline *MSH2* and *MLH1* promoter methylation. ASE analyses of MMR genes were performed in patients with available RNA and heterozygous for at least one allelic marker, independently from the results of the above screenings. Patients negative at the above analyses were tested for *APC* and *MUTYH* sequence variants as described below. The variation data identified in this study have been submitted to the International Society for Gastrointestinal Hereditary Tumours (InSiGHT, http://www.insight-group.org/variants/database/) database.

### Screening for germline nucleotide substitutions in MMR genes

Patients were initially screened for sequence variants in *MSH2* and *MLH1* using single strand conformation polymorphism (SSCP) analysis or denaturing gradient gel electrophoresis (DGGE). All cases negative at SSCP or DGGE were further screened for variants in *MSH2*, *MLH1* and *MSH6* by denaturing high performance liquid chromatography (dHPLC) and automated sequencing, which detected a few additional mutations escaped at the initial screening (data not shown). To predict potential deleterious effects of novel VUS we used the following *in silico* tools: PolyPhen-2 (http://genetics.bwh.harvard.edu/pph2/), SIFT (http://sift.jcvi.org/), HSF (http://www.umd.be/HSF/), FruitFly (http://www.fruitfly.org/seq_tools/splice.html), Mutation Taster (http://www.mutationtaster.org/), Alamut (http://www.interactive-biosoftware.com). We also used MAPP-MMR (http://mappmmr.blueankh.com/) and PON-MMR (http://bioinf.uta.fi/PON-MMR/) prediction algorithms that were specifically validated for missense variants in MMR genes.

### Analysis of extended germline rearrangements in *MSH2, MLH1* and *EPCAM*

Genomic *MSH2* and *MLH1* rearrangements were screened by multiplex ligation-dependent probe amplification (MLPA) in 78 patients negative at initial analysis for pathogenic sequence variants or with VUS. Confirmation of rearrangements was obtained by non-fluorescent multiplex PCR coupled to HPLC (NFMP-HPLC) or by lab-on-a-chip technology for the analysis of copy number variations (LOC-CNV), as previously described [28,29]. Since the original version of the *MSH2/MLH1* MLPA kit (SALSA P003, MRC-Holland, Amsterdam, The Netherlands) employed did not include probes corresponding to *EPCAM*, we developed 3 novel NFMP-HPLC assays for screening and confirmation of genomic rearrangements affecting the 3’ end of *EPCAM* and the intergenic *MSH2*-*EPCAM* region (Table S1). These assays were performed in a subset of 33 cases with VUS or negative at previous screenings and for whom DNA had not been used up in previous analyses. Breakpoint analysis was performed as previously described [28,29].

### Germline *MSH2* and *MLH1* promoter methylation

Bisulphite DNA conversion was performed in in 44 cases negative at initial screening for pathogenic sequence variants and genomic rearrangements using the Imprinting DNA modification kit (SIGMA, Saint Louis, MO), according to the manufacturer's instructions. Screening for germline *MLH1* promoter methylation was conducted by methylation-specific PCR (MSP) using a degenerate and a methylation-specific primer designed by Suter et al [30]. Furthermore, we designed a control PCR in which the degenerate primer used for MSP was paired to a primer specific for unmethylated DNA (Table S2). DNA from the SW48 cell line was used as positive *MLH1* promoter methylation control. Screening for germline *MSH2* promoter methylation was conducted by MSP using primers specific for methylated and unmethylated alleles, as described by Chan et al [31]. Positive controls for *MSH2* promoter methylation were obtained using control DNAs that had been universally methylated using S-adenosylmethionine (SAM) and M.SssI CpG methyltrasferase (New England BioLabs, Ipswich, MA).

### ASE analysis

ASE analyses of *MSH2*, *MLH1* or *MSH6* were performed by primer extension in patients with available RNA and heterozygous for at least one allelic marker, independently from their mutational status. ASE analyses were carried out as previously described using either ^32^P-labeled or unlabeled primers coupled to analysis by Molecular Imager (Bio Rad Laboratories, Hercules, CA) or by DHPLC (Transgenomic, Omaha, NE), respectively [23,25]. Three ASE assays were performed both with ^32^P-labeled primers and with the DHPLC-based method, which yielded comparable results (Table S3). For each ASE assay, the mean ratio obtained with gDNA templates was employed to normalize the data generated in primer extension experiments conducted using cDNA templates. These normalized cDNA/gDNA ratios were designated as ASE values. Based on previous studies, only marked imbalances in relative allele expression corresponding to twofold imbalances in allelic ratios (<1:2 or >2:1 ratios, equivalent to ASE values <0.5 or >2, respectively) were conservatively considered evidence of a pathogenic alteration [23,25,26]. These ASE values deviate more than 3 SDs from mean values observed in heterozygous controls for two CRC predisposing genes that we have previously analyzed based on the availability of ASE markers frequent in the Italian population (ASE of *MLH1* in controls, mean 1.04, SD 0.11, using rs1799977; ASE of *APC* in controls, mean 1.25, SD 0.21, using rs2229992) [24,25]. 

 Overall, ASE in *MLH1*, *MSH2* and *MSH6* was measured using 8 previously described [23,25] and 3 novel assays. Primers for novel assays are described in Table S4.

### Tumor MSI and IHC analyses

Tumor assays were performed at the collaborating institutions that recruited the patients. MSI was analyzed in most of the probands (93/102) with available tumor samples, according to international guidelines [32]. Immunohistochemical expression of MSH2, MLH1 and MSH6 proteins was analyzed for 73/102 cases with available tumor samples as previously described [13].

### Screening for *APC* and *MUTYH* nucleotide substitutions

The coding sequence and intron-exon borders of *APC* and *MUTYH* were analyzed by direct sequencing in 19 probands negative at MMR gene screening. These patients were selected based on availability of DNA and on the presence of at least one polyp at diagnosis in the probands and/or in their relatives.

## Results

Multiple approaches were used to analyze 132 unrelated HNPCC patients for pathogenic defects in MMR and non-MMR genes. 

### MSH2, MLH1 and MSH6 nucleotide variants

We detected 41 previously described and 15 novel MMR gene variants (Table 1 and Table S5). These included 27 loss-of-function nucleotide changes (nonsense and frameshift) introducing premature termination codons (PTCs), 14 variants located at splice sites, 3 in-frame deletions and 12 missense substitutions. Eight of the splice site variants were experimentally verified to associate with altered splicing, including 7 analyzed in previous studies [33-38] and 1 in the present study (see below), while 5 were considered pathogenic being located at the almost invariant dinucleotides at intron ends (Table 1 and Table S5). Two in-frame deletions and 9 missense variants were reported as pathogenic or potentially pathogenic based on *in silico* analyses, functional assays, qualitative classifier or multifactorial prediction model in previous reports [39-44], as specified in Table S5.

**Table 1:** Overview of MSI, IHC and mutational status in 132 HNPCC unrelated patients meeting AC.

**Patients**	**AC**	**MSI**	**MMR defective IHC**	**Germline defects^*a*^**	
				**nucleotide variants and rearrangements**	**altered ASE^*b*^**
LCH-1	I	MSI-H	MLH1	*MLH1* c.301GA (p.Gly101Ser)	
GDLM-2#III-1	I	MSI-H	MSH2	*MSH2* c.942+3AT	
GDLM-7#III-3	I	MSI-H	MLH1/MSH2	*MSH2* Del exon 7	
LCH-8	I	MSI-H	MSH2		
GDLV-11#II-9	I	MSI-H	MLH1		
96#1636	II	MSI-H	MLH1		
GDLM-9#II-2	I	MSI-H	MSH2	*MSH2* c.1549_1550delGCinsT (p.Ala517Tyrfs*9)	*MSH2*
GDLG-18#III-19	I	MSI-H	n.i.	*MSH2* Del exon 3	
LCH-19	I	MSI-H	MSH2	*MSH2* c.2245GT (p.Glu749*)	
GDLG-20#II-1	I	MSI-H	MLH1		*MLH1*
LCH-27	I	MSI-H	MSH2		*MLH1*
GDLG-49#IV-2	I	MSI-H	MSH2	*MSH2* c.1024GA (p.Val342Ile)	
GDLV-52#II-2	I	MSI-H	MLH1		*MLH1*
LCH-57	I	MSI-H	MLH1	*MLH1* c.1989GT (p.Glu663Asp)	
LCH-58	I	MSI-H	MSH2	*MSH2* c.2005+3_2005+14del12	
LCH-59	I	MSI-H	MSH2	*MLH1* c.1679delT (p.Phe560Serfs*31)	
LCH-88	I	MSI-H	n.a.	*MLH1* c.1731GA (p.Ser577Ser)	
LCH-93	II	MSI-H	n.a.	*MSH2* c.1046CG (p.Pro349Arg)	
19#719	II	MSI-H	MSH2	*MSH2* c.1444dupA (p.Arg482Lysfs*6)	
297#875	I	MSI-H	MLH1	*MLH1* c.1852_1854delAAG (p.Lys618del)	
307#2619	I	MSI-H	MLH1	*MLH1* c.199GA (p.Gly67Arg)	
309#3478	I	MSI-H	MSH2	*MSH2* Del exons 9-10	
311#2042	I	MSI-H	n.i.	*MLH1* c.731GA (p.Gly244Asp)	
338#1489	I	MSI-H	MLH1/MSH6	*MLH1* c.382GC (p.Ala128Pro)	
349#1581	I	MSI-H	MSH2	*MSH2* c.942+3AT	
363#2541	I	MSI-H	MSH2	*MSH2* Del exons 9-10	
412#3342	II	MSI-H	MSH2	*EPCAM* Del exon 3 *- MSH2* exon 7	
459#2809	I	MSI-H	MSH2	*MSH2* Del 5'upstream region - exon 8	
476#R26	I	MSI-H	MSH2	*MSH2* Del exons 1-6	
667#2412	I	MSI-H	MSH2	*MSH2* c.942+3AT	
668#2371	I	MSI-H	MLH1	*MLH1* c.545+3AG	
670#2413	I	MSI-H	MLH1	*MLH1* c.1989GT (p.Glu663Asp)	
727#AA	I	MSI-H	MSH2	***MSH2* c.942+2TA**	
814#DGR	I	MSI-H	n.a.	***MSH2* c.[376GγA(;)2251G>C**]** (p**.[**Gly126Ser**(;)**Gly751Arg**])	
986#3487	I	MSI-H	MLH1	***MLH1* c.1731+4AG**	
1068#3015	I	MSI-H	MLH1	*MLH1* c.1050delA (p.Gly351Aspfs*16)	
1070#2957	I	MSI-H	MSH2	***MSH2* c.2287G>C (p.Ala763Pro)**	
1080#2974	II	MSI-H	n.a.	*MSH2* c.2089CT (p.Cys697Arg)	
1251#3260	II	MSI-H	MSH2	*MSH2* c.374C>T (p.Gln125*)	
1256#3479	II	MSI-H	MSH2	*MSH2* c.1786_1788delAAT (p.Asn596del)	
1293#3286	I	MSI-H	MLH1	*MLH1* Del exon 6	
1301#3323	II	MSI-H	MSH2	***MSH2* c.2519_2530del12 (p.Val840_Cys843del)**	
1459#3324	II	MSI-H	MLH1	*MLH1* c.1852_1854delAAG (p.Lys618del)	
LES1#LP	II	MSI-H	n.a.	*MLH1* c.731GA (p.Gly244Asp)	
298#668/1584	I	MSI-H	MSH2	*MSH2* c.1077-2A>C	
319#1004	I	MSI-H	MSH2	*MSH2* c.1046CG (p.Pro349Arg)	
350#1933	I	MSI-H	MLH1	*MLH1* c.676CT (p.Arg226*)	
360#2916	I	MSI-H	MLH1/MSH6	***MLH1* c.1731+4AG**	*MLH1*
903#2630	II	MSI-H	MSH2	*MSH2* Del exon 3	
1218#3238	I	MSI-H	MLH1	*MLH1* Dup exons 2-3	
1515#3442	II	MSI-H	MSH2	*MSH2* c.1255CT (p.Gln419*)	
334#1170	I	MSI-H	MSH2	*MSH2* c.278_279delTT (p.Leu93Profs*6)	*MSH2*
711#2495	II	MSI-H	MLH1	*MLH1* c.1459CT (p.Arg487*)	
1205#BA	II	MSI-H	MLH1	*MSH2* c.1215CA (p.Tyr405*)	
1206#GE	I	MSI-H	MSH2	*MSH2* c.1046CG (p.Pro349Arg)	
357#2038	I	MSI-H	n.a.	*MSH2* c.2294delC (p.Ala765Valfs*47)	
600#2237	I	MSI-H	n.a.	*MSH2* Del exons 4-6	
1138#3149	I	MSI-H	n.a.	*MLH1* c.1852_1854delAAG (p.Lys618del)	
737#2838	I	MSI-H	n.a.	*MLH1* c.1989GT (p.Glu663Asp)	
TO9726	I	MSI-H	n.a.	*MLH1* c.2040CA (p.Cys680*)	
GE9804	I	MSI-H	n.a.	*MSH2* c.1705_1706delGA (p.Glu569Ilefs*2)	
GE9726	I	MSI-H	n.a.	*MSH2* c.2536CT (p.Gln846*)	
SI9744	I	MSI-H	n.a.	*MSH2* c.942+3AT	
GE9914	I	MSI-H	n.a.	*MLH1* c.677+1GA	
F102	I	MSI-H	n.a.	*MLH1* c.546-2A>G	
SI9606	I	MSI-H	n.a.	*MLH1* c.911AT (p.Asp304Val)	
LCH-4	I	MSI-H	n.i.		
LCH-12	I	MSI-H	n.i.		
LCH-85	I	MSI-H	n.a.		
LCH-47	I	MSI-H	n.a.		
871#R08	I	MSI-H	n.a.		
GE0101	I	MSI-H	n.a.		
GE9801	I	MSI-H	n.a.		
GE9903	II	MSI-H	n.a.		
LCH-6	I	MSS	n.a.		
LCH-13	I	MSS	n.i.		
LCH-79	I	MSS	n.a.		
296#776	I	MSS	n.i.		
303#2547	I	MSS	n.i.		
308#1260	I	MSS	n.i.	*MUTYH* c.[1145G>A];[1395_1397delGGA] (p.[(Gly382Asp)];[(Glu466del)]	
313#1381	I	MSS	n.a.		
342#2803	I	MSS	n.i.		
365#2192	II	MSS	n.a.		
368#2506	I	MSS	n.i.		
369#2169	I	MSS	n.a.		
370#3105	I	MSS	n.i.	*APC* c.1100_1101delCT (p.Ser367Cysfs*10)	
633#3114	I	MSS	n.i.		
728#2509	I	MSS	n.i.		
818#2543	I	MSS	n.i.		
933#R14	I	MSS	n.a.		
1165#3159	I	MSS	n.i.		
1193#3187	I	MSS	n.i.		
SV0001	I	MSI-L	n.a.		
TO9913	I	n.a.	MLH1		
705#3035	I	n.a.	MLH1	*MLH1* c.1367C>A (p.Ser456*)	
1008#2829	I	n.a.	MSH2	*MSH2* c.1757C>G (p.Ser586*)	
1077#2979	I	n.a.	MLH1	***MLH1* c.453+1GA**	
1420#3343	II	n.a.	MSH2	*MSH2* c.868GT (p.Glu290*)	
LCH-15	I	n.a.	n.i.	*MLH1* c.1918CT (p.Pro640Ser)	
LCH-23	II	n.a.	n.a.	*MSH2* Del exon 1	
GDLG-29#III-8	II	n.a.	n.a.	*MLH1* c.1852_1854delAAG (p.Lys618del)	
GDLG-31#III-11	I	n.a.	n.a.	*MLH1* c.954delC (p.His318Glnfs*49)	*MLH1*
LCH-86	I	n.a.	n.a.	*MSH2* c.212-1G>A	
83#3103	I	n.a.	n.a.	*MLH1* Del exon1	*MLH1*
601#2307	I	n.a.	n.a.	*MSH6* c.3013CT (p.Arg1005*)	
1157#834	I	n.a.	n.a.	*MSH2* c.119delG (p.Gly40Alafs*24)	
324#R10	I	n.a.	n.a.	*MLH1* c.1896GA (p.Glu632Glu)	
337#2224	I	n.a.	n.a.	*MLH1* c.1989GT (p.Glu663Asp)	
359#2578	I	n.a.	n.a.	*MLH1* c.1639_1643dupTTATA (p.Leu549Tyrfs*44)	
463#3031	I	n.a.	n.a.	*MLH1* c.1852_1854delAAG (p.Lys618del)	
602#2416	I	n.a.	n.a.	*MLH1* c.1011delC (p.Asp338Ilefs*29)	
985#2683	I	n.a.	n.a.	*MLH1* Del exon 6	
1074#3001	I	n.a.	n.a.	*MSH2* c.1059delG (p.Asn354Thrfs*3)	
1200#3221	I	n.a.	n.a.	*MSH6* c.738_741delAAAA (p.Lys246Asnfs*32)	
GE0201	I	n.a.	n.a.	*MSH2* Del exon16	
GE9911	I	n.a.	n.a.	*MLH1* c.1011delC (p.Asp338Ilefs*29)	
TO0012	I	n.a.	n.a.	*MLH1* c.1888_1892delATTGA (p.Ile630*)	
TO0225	I	n.a.	n.a.	*MLH1* c.2040CA (p.Cys680*)	
GE0410	I	n.a.	n.a.	*MSH2* c.942+3AT	
GE0330	I	n.a.	n.a.	*MSH2* c.1216CT (p.Arg406*)	
162#2696	I	n.a.	n.a.	*MLH1* c.1559-2A>G	
LCH-10	I	n.a.	n.i.		
LCH-16	I	n.a.	n.a.		
LCH-51	I	n.a.	n.a.		
LCH-53	I	n.a.	n.a.		
54#982	I	n.a.	n.i.		
314#1200	I	n.a.	n.i.		
353#1114	I	n.a.	n.a.		
455#2186	I	n.a.	n.a.		
1082#2982	I	n.a.	n.a.		
F435	I	n.a.	n.a.		
GE0002	I	n.a.	n.a.		

na, data not available; ni, IHC not informative.

^a^ Novel variants are in bold. Germline MMR gene defects for some patients have been previously reported (see Table 2 and Tables S3, S5, S6 and S7). We also detected several variants previously reported as nonpathogenic (see Table S5).

^b^ ASE value <0.5 or >2.

We performed *in silico* analyses to infer the functional effect of 4 variants (3 missense and 1 intronic) for which no such information was available from previous reports. For the 3 novel missense variants a deleterious effect on protein function was predicted by *in silico* tools including PON-MMR and MAPP-MMR (Table S5). *In silico* tools indicated a potential effect on splicing for the novel intronic variant (*MLH1* c.1731+4AG) (see below) (Table S5). An additional variant, the novel *MSH2* in-frame deletion (c.2519_2530del12, p.Val840_Cys843del), was considered potentially pathogenic because it removes 4 amino acid residues highly conserved in other species and is located in the ATPase domain of the MSH2 protein, where variations were previously reported to cause defects in mismatch binding or release [45,46].

One patient (814#/DGR) was found to carry two missense substitutions in *MSH2* (c.376GA and c.2251GC) predicted to be pathogenic (see Table S5). Unfortunately, no relatives of this patient were available for gene testing and the phase of the two variants could not be ascertained. 

Overall, screening for sequence variants in HNPCC related genes allowed the detection of 56 different substitutions with a definite or potential pathogenic significance in 72 patients, including 26 in *MLH1* (46.5%), 28 in *MSH2* (50%) and 2 in *MSH6* (3.5%) (Table 1 and Table S5).

### Genomic rearrangements of MSH2, MLH1 and EPCAM

Screening of *MLH1* and *MSH2* by MLPA, followed by confirmatory tests using LOC-CNV and/or NFMP-HPLC, indicated the presence of genomic rearrangements (including 14 deletions and 1 duplication) in 15 of the 78 patients analyzed (19%) (Table 1 and Table S6). The gene affected by the rearrangement was *MSH2* in 10 probands (13%) and *MLH1* in 5 probands (6.5%). *EPCAM* rearrangements were screened by NFMP-HPLC in patients without detectable pathogenic variants or with VUS. Furthermore, *EPCAM* rearrangements were evaluated in 3 patients (476#R26, 459#2809 and 412#3342) that based on MLPA and NFMP-HPLC screening had evidence of *MSH2* deletions involving the first exon of the gene (Table S6). One of these patients (476#R26) had a deletion of *MSH2* exons 1-6 and NFMP-HPLC-based EPCAM assays indicated that the rearrangement did not extend to the *EPCAM-MSH2* intergenic region (Table S6). In another patient (459#2809), previously reported to have a deletion spanning exons 1-8 of *MSH2* [28], EPCAM assays showed that the deletion included the *MSH2* 5’ upstream region, but spared the 3’ end of *EPCAM* (Figure S1). In the third patient (412#3342) the deletion affected all *EPCAM* exons included in the EPCAM assays (exons 3, 8 and 9) (Figure S1). This deletion extended up to exon 7 of *MSH2*, as indicated by MLPA and NFMP-HPLC (Table S6). No *EPCAM* rearrangements were detected in the other patients analyzed. All *MLH1, MSH2* and *EPCAM* rearrangements were confirmed by at least 2 independent assays based on MLPA, NFMP-HPLC or LOC-CNV. In 9 cases confirmation of the rearrangements could be also obtained by breakpoint analysis or RT-PCR (Table S6), as previously reported [28,29].

### Germline promoter methylation

Germline MMR gene promoter methylation was analyzed in cases negative at initial screening for pathogenic nucleotide variants and genomic rearrangements. CpG promoter methylation was observed only with positive control DNAs (SW48 cell line for *MLH1* and universally methylated control DNA for *MSH2*, respectively – data not shown). 

### Germline ASE analysis

**Figure 1:**
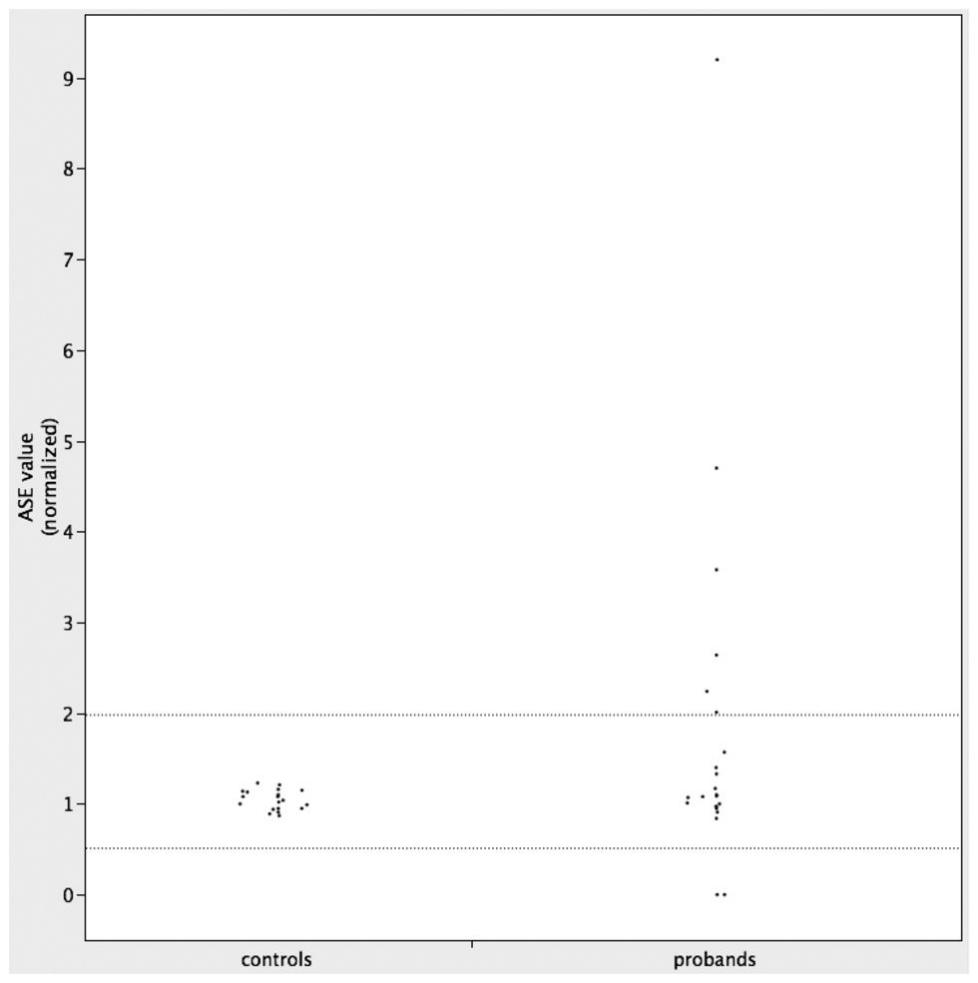
ASE values observed. ASE values between the 2 dashed lines correspond to less than twofold imbalances in allelic ratios (see Methods).

Among the 22 patients that could be analyzed, we observed altered germline ASE of *MLH1* in 6 patients and of *MSH2* in 2 patients (Table 2 and Table S7). Of these patients, 2 showed monoallelic expression of *MLH1* (GDLV-52#II-2 and 83#3103) and 6 had markedly imbalanced expression of *MLH1* (360#2916, GDLG-20#II-1, GDLG-31#III-11 and LCH-27, mean ASE values 4.7, 2.01, 2.24 and 2.64, respectively) or *MSH2* (GDLM-9#II-2 and 334#1170, mean ASE values 3.58 and 9.20, respectively). The remaining patients had modestly imbalanced or balanced ASE (Table 2 and Table S7). ASE values observed in the 22 probands are depicted in Figure 1. For reference, the figure depicts also the values that we have previously measured in control individuals heterozygous for the frequent c.655AG variant (rs1799977) of *MLH1* [25].

**Table 2:** Results of primer extension ASE analyses performed in this study.

**Patients^*a*^**	**Pathogenic germline variants**	**ASE analysis**			
		**Gene**	**ASE marker**	**Consequence**	**Normalized ASE value (SE)^*b*^**
GDLM-2#III-1	*MSH2* c.942+3AT	*MLH1*	c.655AG	p.Ile219Val	1.00 (+0.07)
LCH-8		*MSH2*	c.984CT	p.(=)	1.09 (+0.07)
		*MLH1*	c.655AG	p.Ile219Val	0.97 (+0.16)
GDLG-18#III-19	*MSH2* Del exon 3	*MLH1*	c.655AG	p.Ile219Val	0.91 (+0.10)
LCH-27		*MLH1*	c.655AG	p.Ile219Val	**2.64 (+0.38)**
GDLG-31#III-11	*MLH1* c.954delC (p.His318Glnfs*49)	*MLH1*	c.655AG	p.Ile219Val	**2.24 (+0.07)**
LCH-51		*MLH1*	c.655AG	p.Ile219Val	0.84 (+0.10)
LCH-59	*MLH1* c.1679delT (p.Phe560Serfs*31)	*_MLH1_*	c.655AG	p.Ile219Val	0.97 (+0.11)
GE9804	*MSH2* c.1705_1706delGA (p.Glu569Ilefs*2)	*MLH1*	c.655AG	p.Ile219Val	1.09 (+0.05)
96#1636		*MLH1*	c.655AG	p.Ile219Val	1.17 (+0.06)
334#1170	*MSH2* c.278_279delTT (p.Leu93Profs*6)	*MSH2*	c.278_279delTT	p.Leu93Profs*6	**9.20 (+3.97)**
359#2578	*MLH1* c.1639_1643dupTTATA (p.Leu549Tyrfs*44)	*MLH1*	c.655AG	p.Ile219Val	1.33 (+0.04)
314#1200		*MLH1*	c.655AG	p.Ile219Val	1.01 (+0.03)
1082#2982		*MLH1*	c.655AG	p.Ile219Val	1.07 (+0.02)
		*MSH6*	c.540TC	p.(=)	1.10 (+0.12)
83#3103**^*c*^**	*MLH1* Del exon 1	*MLH1*	c.655AG	p.Ile219Val	**Loss of G allele**

^a^ In Table S7 we summarize the results of ASE analyses for additional 8 patients included in the present study, whose ASE results had been previously reported [23,25].

^b^ Markedly imbalanced ASE values are in bold.

^c^ For this patient PE analysis performed in the present study confirmed the loss of expression for the G allele previously shown by cDNA sequencing [28].

Three patients with altered germline ASE (GDLG-31#III-11, 83#3103 and 334#1170) were known carriers of *MLH1* or *MSH2* deletions. Conversely, in 2 patients with imbalanced ASE initial screening for pathogenic variants by SSCP or DGGE was negative, but subsequent re-analysis by DHPLC and sequencing identified a frameshift of *MSH2* (case GDLM-9#II-2) and an intronic *MLH1* variant with a potential effect on splicing (case 360#2916, see below) (Table 1 and Table S7). Overall, variants with a clear or potential pathogenic role were detected in 5 of the 8 patients with imbalanced ASE (Table 1 and Table S7), whereas in 3 patients (GDLG-20#II-1, LCH-27 and GDLV-52#II-2) altered ASE was the only germline defect detected [23,25].

### Analysis of a novel putative splicing variant

*In silico* analysis by different splice prediction software tools (see Methods) indicated that the effect of the novel *MLH1* c.1731+4AG variant shared by 2 probands (360#2916 and 986#3487) was uncertain. However, they suggested that this change might affect splicing by decreasing the strength of the donor site (average decrease 13%, range 7.4-22%). The availability of cDNA in patient 360#2916 allowed us to test whether the c.1731+4AG variant was associated to a splicing defect. This alteration was elusive at initial testing of RT-PCR amplified cDNA because only wildtype transcripts were revealed by direct sequencing or by electrophoretic analysis (data not shown). However, based on the results of ASE analysis showing a marked imbalance in allelic expression (average normalized allelic ratio 4.7, derived from radioactive and DHPLC-based assays, Table S3), we hypothesized that an altered splicing might be revealed by DHPLC. As predicted, the more sensitive DHPLC analysis allowed the detection of a major peak with a retention time corresponding to the wildtype transcript and of a minor peak corresponding to a less expressed shorter transcript. Sequencing confirmed that the less expressed *MLH1* transcript carried an out-of-frame exon 15 skipping (Figure S2). 

### Tumor MSI and IHC

Among the 93 cases screened for MSI (Table 1), 74 cases (79.5%) were MSI-H and 19 cases were MSS or MSI-L (20.5%). Germline MMR gene defects, including sequence variants, genomic rearrangements or altered ASE were identified in most cases (63/74, 85%) with MSI-H tumors (Table 1). No germline MMR gene alteration was detected in MSS or MSI-L cases.

MLH1, MSH2 or MSH6 protein immunohistochemistry was available for 73 cases and loss of at least one MMR protein was detected in 53 cases (72.6%). Lack of expression affected MLH1 in 21 cases and MSH2 in 29 cases. Moreover, in 1 case (GDLM-7#III-3) IHC showed the loss of both MLH1 and MSH2, whereas in other 2 cases (338#1489 and 360#2916) IHC indicated the loss of both MLH1 and MSH6. We observed discrepancies between the results of IHC and those of other analyses in 12 patients. Among the 20 cases displaying normal MMR protein expression by IHC, 2 cases (LCH-4 and LCH-12) displayed tumor MSI-H phenotype, one case (GDLG-18#III-19) carried a germline deletion of *MSH2* exon 3 and 2 cases (311#2042 and LCH-15) carried missense *MLH1* variants (c.731GA and c.1918CT, respectively) previously reported as potentially pathogenic based on functional or *in silico* analyses, respectively (Table 1) [41,42]. Furthermore, in 2 patients (LCH-59 and LCH-27) IHC indicated the loss of MSH2 expression, but molecular screening revealed germline *MLH1* defects, including a frameshift and a markedly imbalanced ASE, respectively (Table 1). In one additional patient (1205#BA) IHC indicated the loss of MLH1 expression, whereas sequencing revealed a nonsense variant in *MSH2* (Table 1 and Table S5). Finally, in 4 cases IHC indicated the loss of either MSH2 (patient LCH-8) or MLH1 (patients GDLV-11#II-9, 96#1636 and TO9913), but no pathogenic variants in MMR genes were detected.

### APC and MUTYH screening in probands negative for germline MMR defects

Germline defects in polyposis-associated genes were detected in 2 of the 19 patients screened for *APC* and *MUTYH* (Table 1). One patient (370#3105) with an unspecified number of adenomas carried the c.1100_1101delCT variant of *APC*, in the alternatively spliced portion of exon 9. The same nucleotide substitution had been previously reported to associate with attenuated polyposis phenotypes displaying less than 10 adenomas [47]. Another patient (308#1260), with recurrent adenomas at endoscopic follow-up, was a compound heterozygote for the c.1145GA (p.Gly382Asp) and the c.1395_1397delGGA (p.Glu466del) variants of *MUTYH* that were previously reported as pathogenic [13].

### Overall results of germline integrative analyses

**Figure 2:**
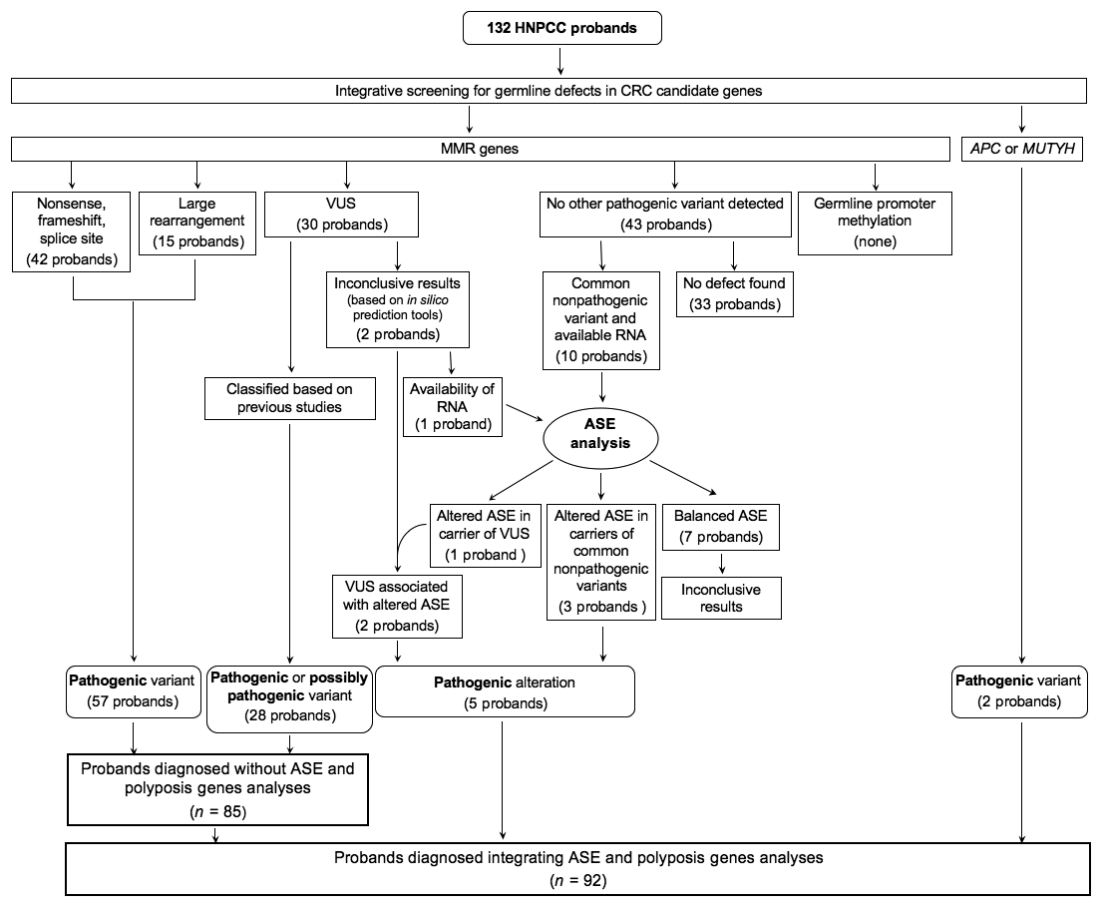
Results of integrative germline analyses performed in this study. VUS included in-frame deletions, sequence variants with uncertain effect on splicing and missense variants. Evidence of a pathogenic, possibly pathogenic defect in 28 probands with VUS derived from previous studies (see Table S5). In one additional case (360#2916) availability of RNA allowed ASE analysis that helped to characterize a VUS shared also by another proband (986#3487, see Results). ASE analysis was conducted in 22 individuals, including 12 with ascertained germline defect that are not shown in the figure. *APC* and MUTYH screening was conducted in a subset of patients negative for MMR defects (see Methods). Inclusion of ASE analysis and screening for *APC* and MUTYH sequence variants in the integrative analyses increased the number of probands with germline alterations detected.

The overall results of integrative germline analyses performed in this study are summarized in Figure 2. Nucleotide variants or rearrangements of MMR genes with a definite pathogenic role were identified respectively in 42 (45.7%) and 15 (16.3%) of the 92 patients with ascertained germline alterations. VUS classified as pathogenic or potentially pathogenic based on previous studies (see Table S5) were identified in 28 (30.4%) of the probands with ascertained germline defects. None of the probands tested showed germline *MSH2* or *MLH1* promoter methylation. Overall, the above analyses identified pathogenic variants in 85 (92.4%) of the probands with ascertained germline alterations. ASE allowed the detection of pathogenic alterations in 5 (5.4%) additional probands, including 3 negative at other screenings and 2 bearing a VUS that caused decreased expression of the corresponding allele. Furthermore, *APC* and *MUTYH* screening allowed the detection of pathogenic variants in 2 (2.2%) additional probands.

## Discussion

In this study we summarize the results of extensive analyses conducted in 132 AC probands providing insights that may help clinical translation of genetic testing in HNPCC. 

In line with other studies [3-5], the majority of the germline MMR gene defects detected were nucleotide variants or rearrangements in *MSH2* or *MLH1*. Among MMR genes less commonly mutated in HNPCC, pathogenic *MSH6* variants were identified only in 2 probands. We did not analyze either genomic rearrangements of *MSH6*, which account for 5% (17/320) of the variants of this gene reported in the June 2013 release of the Human Gene Mutation Database [48], or its germline methylation status. Furthermore, we did not screen *PMS2* or other genes occasionally mutated in HNPCC. It is possible that a fraction of cases negative at our analyses carries unscreened defects in these genes. A substantial proportion of patients (30/72 showing nucleotide changes in MMR genes, 42%) presented VUS, such as missense variants, in-frame deletions and nucleotide substitutions with uncertain effect on splicing. Characterization of these variants is complex and may require the use of assays that are not routinely available [6]. To facilitate classification of VUS many algorithms have been developed including software tools, such as MAPP-MMR, PON-MMR and CoDP, specifically dedicated to MMR genes [42-44,49-52]. Moreover, the pathogenic potential of many VUS recurring in HNPCC was established in previous studies [33-44]. In this regard, 25 of our 30 probands with VUS carried variants that had been previously reported to be pathogenic or possibly pathogenic (Table S5). The remaining 5 probands carried 5 novel VUS, including 3 missense variants (2 occurring in the same proband) predicted to have a deleterious effect using previously developed *in silico* tools (see Table S5), one in-frame deletion that was considered potentially pathogenic based on previous studies showing the crucial function of the amino acids removed and one intronic variant (occurring in 2 probands) whose deleterious effect on allele expression and splicing was characterized in this study based on the results of ASE analysis, as discussed below. 

One remarkable observation in this study is the high proportion (8/22, 36.5%) of germline defects revealed by ASE analysis in the informative probands analyzed. Notably, in a relevant proportion of cases ASE contributed to identify germline alterations that were not detected by other analyses or that were of uncertain significance (Figure 2). In particular, in 3 of these cases, negative for pathogenic variants or VUS, imbalanced ASE was the only germline MMR gene defect identified (Table 1). The 3 patients showed also independent evidence of MMR defects, as indicated by the presence of tumor MSI-H phenotype. These observations show that ASE analysis has the potential to reveal alterations in germline transcript expression even when these are due to defects that may escape detection with traditional sequencing, MLPA or NGS approaches. Such defects include germline epigenetic allele silencing, or nucleotide changes occurring in genomic regions not comprised in the screening, such as *cis*- or trans-acting variants that alter gene regulation and lower the expression of the corresponding allele. In addition to identifying defects in cases negative at other integrative germline screenings, ASE may provide evidence for the pathogenic potential of VUS helping to characterize these variations, as exemplified in this study by the splice site variant detected in patients 360#2916 and 986#3487, both displaying tumor MSI-H phenotype. For this novel VUS, *in silico* analyses provided inconclusive results, whereas ASE analysis with two different markers readily revealed a marked alteration in allelic expression. ASE also guided the characterization of the mutant transcript, which was initially hindered by its lower expression, allowing the detection of exon skipping. Thus, ASE cost-effectively complements molecular analyses in probands negative at other screenings and may be helpful for the characterization of VUS with potential effects on gene expression that may be detected by gDNA-based methods, both traditional or NGS. In fact, NGS is bound to uncover novel coding and non-coding variants with potential effects on gene expression and testing their functional effect appears currently challenging [53]. In these cases, ASE may represent a practical tool to reveal pathogenic germline alterations in the corresponding genes.

None of the probands tested in our study had germline *MSH2* promoter hypermethylation. This epigenetic modification was previously shown to be heritable and associated to deletions affecting the last exon of *EPCAM*, but sparing the contiguous *MSH2* promoter [8,31]. In line with the lack of germline promoter hypermethylation in our probands, the only patient displaying an *EPCAM* rearrangement in this study had a deletion that removed also the *MSH2* promoter and extended up to exon 7 of this gene. Thus, our results indicate that *EPCAM* rearrangements and *MSH2* promoter hypermethylation were not frequent among the probands tested in the present study. We did not detect germline hypermethylation of the *MLH1* promoter and this finding in our probands belonging to families with vertical transmission is consistent with the non-Mendelian inheritance mostly reported for constitutional *MLH1* epimutations [54,55].

Several studies supported the value of tumor IHC and MSI prescreening and these analyses are included in models for multifactorial classification of VUS pathogenicity [6,19,44,51]. In this study, tumor IHC and MSI were generally concordant with the results of germline analyses, but there were several exceptions that limited their practical value. IHC provided indications useful to target germline mutational screening in 46 of the 73 (63%) cases analyzed. In several of our probands (12/73, 16%) the results of IHC were in contrast with those of germline screening for MMR gene defects and/or tumor MSI analysis. The occurrence of discrepancies between IHC, MSI and germline screening for defects in MMR genes was reported also in previous studies [5,19]. As far as MSI, germline MMR gene defects were detected in the majority of MSI-H cases and no MMR gene pathogenic variants were detected in patients with MSS or MSI-L tumors in our study. However, previous studies showed that patients with MSS or MSI-L tumors may occasionally harbor germline MMR gene defects [5,20]. Moreover, the occurrence of somatic *MLH1* silencing was previously shown to be responsible of inconsistencies between germline and tumor MMR defects [5]. In addition to the potential causes of inconsistencies described in previous studies, we cannot exclude that technical issues, such as sample quality and differences in IHC and MSI procedures and interpretation among centers might be responsible for some of the discrepancies between germline and tumor observed in our study. In any case, the occurrence of false positive or negative results with both IHC and MSI also in previous studies [5,19,20] indicates that these tumor assays should be used with caution to include or exclude probands from screening for germline MMR gene defects. In particular, a germline screening based on the results of IHC analyses obtained in this study would have missed germline defects in 6 probands. Furthermore, with the advent of cost-efficient NGS technologies, allowing simultaneous analysis of several CRC predisposing genes, IHC and MSI prescreening may become unnecessary. On the other hand, tumor assays, such as IHC and MSI, may provide evidences that contribute to multifactorial models for VUS classification, as indicated by previous studies [43,44,52].

One important issue in genetic testing of HNPCC is the considerable proportion of patients negative for MMR gene variants observed in several studies [19-21], including the present. This reflects at least in part limitations of MMR gene screenings that are shared by this and other studies and in part the fact that a significant percentage (up to 40%) of families meeting Amsterdam criteria are FCCTX unrelated to MMR defects [4]. It is noteworthy that in our study 2 of the 19 (~10%) probands negative for MMR gene variants and undergoing mutation screening of *APC* and *MUTYH* carried germline defects in one of these two genes. Thus, despite the limited number of patients screened, this finding highlights the importance of analyzing polyposis-associated genes in families negative for MMR gene alterations with a clinical HNPCC phenotype. In this regard, testing of multiple genes is now facilitated by the use of NGS approaches [22]. On the other hand, despite extensive screening of known CRC predisposition genes by NGS, the genetic basis of most cases negative for germline pathogenic defects in those genes remains elusive, as indicated by a recent study that identified only 6 pathogenic variants and 3 VUS among 31 Lynch or polyposis syndrome patients negative at previous analyses [22]. This observation indicates that NGS approaches focusing on candidate genes improve detection of gene variants, but are not sufficient to achieve a definite genetic diagnosis in all probands and that further studies are necessary to clarify the genetic basis of cases negative for pathogenic defects.

Our results provide a number of indications that may help to improve current diagnostic algorithms for HNPCC. Analysis of several CRC predisposing genes was necessary to reveal germline defects and low-throughput methods are not cost-efficient for this task, especially when IHC results do not help to prioritize germline screening, as it occurred for several probands in this study. The recent development of gDNA-based NGS assays for parallel analysis of CRC predisposing genes (including MMR genes, *APC* and *MUTYH* tested in this study) opens a new possibility for more efficient genetic screening of HNPCC at reasonable costs. This technology has the potential to reveal both sequence variants and rearrangements that are the most frequent pathogenic defects detected in HNPCC. We screened for MMR gene rearrangements using MLPA that may still represent a cost-efficient approach for these relatively frequent defects. Confirmation of putative rearrangements was obtained using low-throughput methods, preferably by direct demonstration of breakpoints. Considering that breakpoint determination may be time-consuming and impractical, indirect methods may be applied, as exemplified by a recent study that confirmed by MLPA the putative rearrangements indicated by NGS [22]. The use of NFMP-DHPLC or LOC-CNV for rearrangement confirmation may represent a cost-effective alternative to MLPA, because these methods have lower analytical costs and assays validated in this or previous studies are already available [28,29]. In our study none of the probands analyzed showed germline hypermethylation of MMR gene promoters. However, when such alterations are identified ASE analysis of the corresponding gene may provide an independent evidence of a pathogenic effect. In this study, ASE analysis helped to characterize one VUS with potential effects on splicing and revealed germline defects in 3 probands where only common nonpathogenic variants were detected. Thus, inclusion of ASE in our integrative screening (Figure 2) contributed to increase the proportion of patients with germline MMR defects identified as compared to previously proposed diagnostic algorithms that do not include ASE [17-21].

In conclusion, this study provides an overview of some relevant issues encountered in the genetic diagnosis of HNPCC using a variety of methods and supports the notion that ASE analysis and *APC* and *MUTYH* screening should be integrated in diagnostic algorithms to improve clinical translation of genetic testing in CRC predisposing syndromes.

## Supporting Information

Figure S1 **NFMP-HPLC assays for EPCAM genomic rearrangements.** Probes for EPCAM-1 and EPCAM-2 assays are indicated based on their position in the region encompassing EPCAM and the 5’upstream region of MSH2 (panel **a**). Examples of EPCAM-1 (panel b) and EPCAM-2 (panel c) profiles are shown for control individuals (top of each panel) and 2 representative patients (459#2809, middle of each panel; 412#3342, bottom of each panel). Control peaks are labeled “c” and arrows indicate amplicons with decreased peak heights, indicative of genomic deletions. EPCAM-1 (panel **b**): in patient 459#2809 the chromatographic profile shows decreased peak heights for the 2 amplicons corresponding to the MSH2 5’ upstream region (proximal and distal); in patient 412#3342 all the *EPCAM-MSH2* amplicons included in the assay show decreased peak heights compared to the control peaks. EPCAM-2 (panel **c**): in patient 459#2809 no alterations in the chromatographic profile are observed, confirming the absence of deletions in the EPCAM amplicons tested; in patient 412#33428 the peaks corresponding to the EPCAM probes show decreased heights compared to the control peaks, confirming the deletion of the EPCAM amplicons tested.
(TIF)

Figure S2 **Molecular analysis of exon skipping in patient 360#2916.** Panel **a**: Location of RT-PCR primers in *MLH1* exons 14 and 17. Panel **b**: DHPLC chomatographic profiles obtained with cDNAs from patient 360#2916 and a control. The chromatogram of patient 360#2916 shows a major peak corresponding to the wt transcript and a minor peak corresponding to a less expressed shorter transcript (average allelic ratio 4.50, derived from 3 independent experiments). Panel **c**: DHPLC profiles derived from PCR amplification of the chromatographic fractions corresponding to the purified wt or shorter transcript. Panel **d**: Sequences corresponding to the two purified peaks (sequences of reverse strands are shown). The longer peak displays the wildtype sequence (top sequence), whereas the shorter peak shows the skipping of exon 15 (bottom sequence).
(TIF)

Table S1 **Primers for NFMP-HPLC analysis of *EPCAM-MSH2* rearrangements.** The first NFMP-HPLC multiplex for *EPCAM* (EPCAM-1) consisted of 7 amplicons, including 2 located in exons 3 and 8 of *EPCAM*, 3 located within the intergenic *MSH2*-*EPCAM* region and 2 reference amplicons corresponding to *MSH2* exon 9 and *MLH1* exon 5. The second NFMP-HPLC multiplex for *EPCAM* (EPCAM-2) consisted of 4 amplicons, including 2 located in exons 8 and 9 of *EPCAM* and 2 reference amplicons corresponding to *MSH2* exon 9 and *MLH1* exon 5. The third NFMP-HPLC multiplex for *EPCAM* (EPCAM-3) differed from EPCAM-2 assay only for the reference amplicon that was located within a copy number invariant region in chromosome 2q36.1. Multiplex PCRs were performed using a touchdown protocol in a total of 23 cycles. Reactions were carried out on a GeneAmp PCR System 2720 thermocycler (Applied Biosystems), in a final volume of 10 µl containing 30-50 ng of template DNA and 0.5 unit of AmpliTaq Gold DNA polymerase (Applied Biosystems).
(DOC)

Table S2 **Primers for analysis of germline *MLH1* promoter methylation.**
(DOC)

Table S3 **Comparison between results of ASE analyses performed using 32P-labeled primers or DHPLC.**
(DOC)

Table S4 **Primers for ASE assays developed in this study.**
(DOC)

Table S5 ***MSH2*, *MLH1* and *MSH6* nucleotide variants detected.**
(DOC)

Table S6 **Analysis of genomic rearrangements.**
(DOC)

Table S7 **Correlations between the results of ASE and other analyses.**
(DOC)

## References

[1] Jasperson KW, Tuohy TM, Neklason DW, Burt RW (2010) Hereditary and familial colon cancer. Gastroenterology 138: 2044-2058. doi:10.1053/j.gastro.2010.01.054. PubMed: 20420945. 20420945 PMC3057468

[2] Burn J, Gerdes AM, Macrae F, Mecklin JP, Moeslein G et al. (2011) Long-term effect of aspirin on cancer risk in carriers of hereditary colorectal cancer: an analysis from the CAPP2 randomised controlled trial. Lancet 378: 2081-2087. doi:10.1016/S0140-6736(11)61049-0. PubMed: 22036019. 22036019 PMC3243929

[3] Wagner A, Barrows A, Wijnen JT, van der Klift H, Franken PF et al. (2003) Molecular analysis of hereditary nonpolyposis colorectal cancer in the United States: high mutation detection rate among clinically selected families and characterization of an American founder genomic deletion of the MSH2 gene. Am J Hum Genet 72: 1088-1100. doi:10.1086/373963. PubMed: 12658575. 12658575 PMC1180263

[4] Lindor NM, Rabe K, Petersen GM, Haile R, Casey G et al. (2005) Lower cancer incidence in Amsterdam-I criteria families without mismatch repair deficiency: familial colorectal cancer type X. JAMA 293: 1979-1985. doi:10.1001/jama.293.16.1979. PubMed: 15855431. 15855431 PMC2933042

[5] Mueller J, Gazzoli I, Bandipalliam P, Garber JE, Syngal S et al. (2009) Comprehensive molecular analysis of mismatch repair gene defects in suspected Lynch syndrome (hereditary nonpolyposis colorectal cancer) cases. Cancer Res 69: 7053-7061. doi:10.1158/0008-5472.CAN-09-0358. PubMed: 19690142. 19690142 PMC2761236

[6] Pineda M, González S, Lázaro C, Blanco I, Capellá G (2010) Detection of genetic alterations in hereditary colorectal cancer screening. Mutat Res 693: 19-31. doi:10.1016/j.mrfmmm.2009.11.002. PubMed: 19931546. 19931546

[7] Vasen HF, Watson P, Mecklin JP, Lynch HT (1999) New clinical criteria for hereditary nonpolyposis colorectal cancer (HNPCC, Lynch syndrome) proposed by the International Collaborative group on HNPCC. Gastroenterology 116: 1453-1456. doi:10.1016/S0016-5085(99)70510-X. PubMed: 10348829. 10348829

[8] Ligtenberg MJ, Kuiper RP, Chan TL, Goossens M, Hebeda KM et al. (2009) Heritable somatic methylation and inactivation of MSH2 in families with Lynch syndrome due to deletion of the 3' exons of TACSTD1. Nat Genet 41: 112-117. doi:10.1038/ng.283. PubMed: 19098912. 19098912

[9] Kovacs ME, Papp J, Szentirmay Z, Otto S, Olah E (2009) Deletions removing the last exon of TACSTD1 constitute a distinct class of mutations predisposing to Lynch syndrome. Hum Mutat 30: 197-203. doi:10.1002/humu.20942. PubMed: 19177550. 19177550

[10] Rumilla K, Schowalter KV, Lindor NM, Thomas BC, Mensink KA et al. (2011) Frequency of deletions of EPCAM (TACSTD1) in MSH2-associated Lynch syndrome cases. J Mol Diagn 13: 93-99. doi:10.1016/j.jmoldx.2010.11.011. PubMed: 21227399. 21227399 PMC3069927

[11] Niessen RC, Hofstra RM, Westers H, Ligtenberg MJ, Kooi K et al. (2009) Germline hypermethylation of MLH1 and EPCAM deletions are a frequent cause of Lynch syndrome. Genes Chromosomes Cancer 48: 737-744. doi:10.1002/gcc.20678. PubMed: 19455606. 19455606

[12] Kloor M, Voigt AY, Schackert HK, Schirmacher P, von Knebel Doeberitz M et al. (2011) Analysis of EPCAM protein expression in diagnostics of Lynch syndrome. J Clin Oncol 29: 223-227. doi:10.1200/JCO.2010.32.0820. PubMed: 21115857. 21115857

[13] Peterlongo P, Mitra N, Sanchez de Abajo A, de la Hoya M, Bassi C et al. (2006) Increased frequency of disease-causing MYH mutations in colon cancer families. Carcinogenesis 27: 2243-2249. doi:10.1093/carcin/bgl093. PubMed: 16774938. 16774938

[14] Morak M, Massdorf T, Sykora H, Kerscher M, Holinski-Feder E (2011) First evidence for digenic inheritance in hereditary colorectal cancer by mutations in the base excision repair genes. Eur J Cancer 47: 1046-1055. doi:10.1016/j.ejca.2010.11.016. PubMed: 21195604. 21195604

[15] Nieminen TT, Abdel-Rahman WM, Ristimäki A, Lappalainen M, Lahermo P et al. (2011) BMPR1A mutations in hereditary nonpolyposis colorectal cancer without mismatch repair deficiency. Gastroenterology 141: e23-e26. doi:10.1053/j.gastro.2011.03.063. PubMed: 21640116. 21640116

[16] Soravia C, DeLozier CD, Dobbie Z, Berthod CR, Arrigoni E et al. (2006) Double frameshift mutations in APC and MSH2 in the same individual. Int J Colorectal Dis 21: 79-83. PubMed: 16676398. 16676398 10.1007/s00384-005-0772-z

[17] Piñol V, Castells A, Andreu M, Castellví-Bel S, Alenda C et al. (2005) Accuracy of revised Bethesda guidelines, microsatellite instability, and immunohistochemistry for the identification of patients with hereditary nonpolyposis colorectal cancer. JAMA 293: 1986-1994. doi:10.1001/jama.293.16.1986. PubMed: 15855432. 15855432

[18] Kievit W, de Bruin JH, Adang EM, Severens JL, Kleibeuker JH et al. (2005) Cost effectiveness of a new strategy to identify HNPCC patients. Gut 54: 97-102. doi:10.1136/gut.2004.039123. PubMed: 15591512. 15591512 PMC1774368

[19] Woods MO, Younghusband HB, Parfrey PS, Gallinger S, McLaughlin J et al. (2010) The genetic basis of colorectal cancer in a population-based incident cohort with a high rate of familial disease. Gut 59: 1369-1377. doi:10.1136/gut.2010.208462. PubMed: 20682701. 20682701 PMC3047452

[20] Lagerstedt Robinson K, Liu T, Vandrovcova J, Halvarsson B, Clendenning M et al. (2007) Lynch syndrome (hereditary nonpolyposis colorectal cancer) diagnostics. J Natl Cancer Inst 99: 291-299. doi:10.1093/jnci/djk051. PubMed: 17312306. 17312306

[21] van Lier MG, Leenen CH, Wagner A, Ramsoekh D, Dubbink HJ et al. (2012) Yield of routine molecular analyses in colorectal cancer patients ≤70 years to detect underlying Lynch syndrome. J Pathol 226: 764-774. doi:10.1002/path.3963. PubMed: 22081473. 22081473

[22] Pritchard CC, Smith C, Salipante SJ, Lee MK, Thornton AM et al. (2012) ColoSeq provides comprehensive Lynch and polyposis syndrome mutational analysis using massively parallel sequencing. J Mol Diagn 14: 357-366. doi:10.1016/j.jmoldx.2012.03.002. PubMed: 22658618. 22658618 PMC3391416

[23] Curia MC, Palmirotta R, Aceto G, Messerini L, Verì MC et al. (1999) Unbalanced germ-line expression of hMLH1 and hMSH2 alleles in hereditary nonpolyposis colorectal cancer. Cancer Res 59: 3570-3575. PubMed: 10446963. 10446963

[24] Curia MC, De Iure S, De Lellis L, Veschi S, Mammarella S et al. (2012) Increased variance in germline allele-specific expression of APC associates with colorectal cancer. Gastroenterology 142: 71-77. doi:10.1053/j.gastro.2011.09.048. PubMed: 21995949. 21995949 PMC3246305

[25] Aceto GM, De Lellis L, Catalano T, Veschi S, Radice P et al. (2009) Nonfluorescent denaturing HPLC-based primer-extension method for allele-specific expression: application to analysis of mismatch repair genes. Clin Chem 55: 1711-1718. doi:10.1373/clinchem.2009.126300. PubMed: 19628660. 19628660

[26] Renkonen E, Zhang Y, Lohi H, Salovaara R, Abdel-Rahman WM et al. (2003) Altered expression of MLH1, MSH2, and MSH6 in predisposition to hereditary nonpolyposis colorectal cancer. J Clin Oncol 21: 3629-3637. doi:10.1200/JCO.2003.03.181. PubMed: 14512394. 14512394

[27] Castellsagué E, González S, Guinó E, Stevens KN, Borràs E et al. (2010) Allele-specific expression of APC in adenomatous polyposis families. Gastroenterology 139: 439-447. doi:10.1053/j.gastro.2010.04.047. PubMed: 20434453. 20434453 PMC2910837

[28] De Lellis L, Curia MC, Catalano T, De Toffol S, Bassi C et al. (2006) Combined use of MLPA and nonfluorescent multiplex PCR analysis by high performance liquid chromatography for the detection of genomic rearrangements. Hum Mutat 27: 1047-1056. doi:10.1002/humu.20386. PubMed: 16941473. 16941473

[29] De Lellis L, Mammarella S, Curia MC, Veschi S, Mokini Z et al. (2012) Analysis of gene copy number variations using a method based on lab-on-a-chip technology. Tumori 98: 126-136. PubMed: 22495713. 22495713 10.1177/030089161209800118

[30] Suter CM, Martin DI, Ward RL (2004) Germline epimutation of MLH1 in individuals with multiple cancers. Nat Genet 36: 497-501. doi:10.1038/ng1342. PubMed: 15064764. 15064764

[31] Chan TL, Yuen ST, Kong CK, Chan YW, Chan AS et al. (2006) Heritable germline epimutation of MSH2 in a family with hereditary nonpolyposis colorectal cancer. Nat Genet 38: 1178-1183. doi:10.1038/ng1866. PubMed: 16951683. 16951683

[32] Boland CR, Thibodeau SN, Hamilton SR, Sidransky D, Eshleman JR et al. (1998) A National Cancer Institute Workshop on Microsatellite Instability for cancer detection and familial predisposition: development of international criteria for the determination of microsatellite instability in colorectal cancer. Cancer Res 58: 5248-5257. PubMed: 9823339. 9823339

[33] Pensotti V, Radice P, Presciuttini S, Calistri D, Gazzoli I et al. (1997) Mean age of tumor onset in hereditary nonpolyposis colorectal cancer (HNPCC) families correlates with the presence of mutations in DNA mismatch repair genes. Genes Chromosomes Cancer 19: 135-142. doi:10.1002/(SICI)1098-2264(199707)19:3. PubMed: 9218993. 9218993

[34] Tanko Q, Franklin B, Lynch H, Knezetic J (2002) A hMLH1 genomic mutation and associated novel mRNA defects in a hereditary non-polyposis colorectal cancer family. Mutat Res 503: 37-42. doi:10.1016/S0027-5107(02)00031-3. PubMed: 12052501. 12052501

[35] Nakagawa H, Yan H, Lockman J, Hampel H, Kinzler KW et al. (2002) Allele separation facilitates interpretation of potential splicing alterations and genomic rearrangements. Cancer Res 62: 4579-4582. PubMed: 12183410. 12183410

[36] Auclair J, Busine MP, Navarro C, Ruano E, Montmain G et al. (2006) Systematic mRNA analysis for the effect of MLH1 and MSH2 missense and silent mutations on aberrant splicing. Hum Mutat 27: 145-154. doi:10.1002/humu.20280. PubMed: 16395668. 16395668

[37] Wijnen J, Khan PM, Vasen H, Menko F, van der Klift H et al. (1996) Majority of hMLH1 mutations responsible for hereditary nonpolyposis colorectal cancer cluster at the exonic region 15-16. Am J Hum Genet 58: 300-307. PubMed: 8571956. 8571956 PMC1914526

[38] Froggatt NJ, Joyce JA, Davies R, Gareth D, Evans R et al. (1995) A frequent hMSH2 mutation in hereditary non-polyposis colon cancer syndrome. Lancet 345: 727. doi:10.1016/S0140-6736(95)90900-1. PubMed: 7885145. 7885145

[39] Heinen CD, Wilson T, Mazurek A, Berardini M, Butz C et al. (2002) HNPCC mutations in hMSH2 result in reduced hMSH2-hMSH6 molecular switch functions. Cancer Cell 1: 469-478. doi:10.1016/S1535-6108(02)00073-9. PubMed: 12124176. 12124176

[40] Gammie AE, Erdeniz N, Beaver J, Devlin B, Nanji A et al. (2007) Functional characterization of pathogenic human MSH2 missense mutations in Saccharomyces cerevisiae. Genetics 177: 707-721. doi:10.1534/genetics.107.071084. PubMed: 17720936. 17720936 PMC2034637

[41] Takahashi M, Shimodaira H, Andreutti-Zaugg C, Iggo R, Kolodner RD et al. (2007) Functional analysis of human MLH1 variants using yeast and in vitro mismatch repair assays. Cancer Res 67: 4595-4604. doi:10.1158/0008-5472.CAN-06-3509. PubMed: 17510385. 17510385

[42] Chao EC, Velasquez JL, Witherspoon MS, Rozek LS, Peel D et al. (2008) Accurate classification of MLH1/MSH2 missense variants with multivariate analysis of protein polymorphisms-mismatch repair (MAPP-MMR). Hum Mutat 29: 852-860. doi:10.1002/humu.20735. PubMed: 18383312. 18383312

[43] Thompson BA, Greenblatt MS, Vallee MP, Herkert JC, Tessereau C et al. (2013a) Calibration of Multiple In Silico Tools for Predicting Pathogenicity of Mismatch Repair Gene Missense Substitutions. Hum Mutat 34: 255-265. doi:10.1002/humu.22214. PubMed: 22949387. 22949387 PMC4318556

[44] Thompson BA, Goldgar DE, Paterson C, Clendenning M, Walters R et al. (2013b) A Multifactorial Likelihood Model for MMR Gene Variant Classification Incorporating Probabilities Based on Sequence Bioinformatics and Tumor Characteristics: A Report from the Colon Cancer Family Registry. Hum Mutat 34: 200-209. doi:10.1002/humu.22213. 22949379 PMC3538359

[45] Lützen A, de Wind N, Georgijevic D, Nielsen FC, Rasmussen LJ (2008) Functional analysis of HNPCC-related missense mutations in MSH2. Mutat Res 645: 44-55. doi:10.1016/j.mrfmmm.2008.08.015. PubMed: 18822302. 18822302

[46] Ollila S, Dermadi Bebek D, Jiricny J, Nyström M (2008) Mechanisms of pathogenicity in human MSH2 missense mutants. Hum Mutat 29: 1355-1363. doi:10.1002/humu.20893. PubMed: 18951462. 18951462

[47] Curia MC, Esposito DL, Aceto G, Palmirotta R, Crognale S et al. (1998) Transcript dosage effect in familial adenomatous polyposis: model offered by two kindreds with exon 9 APC gene mutations. Hum Mutat 11: 197-201. doi:10.1002/(SICI)1098-1004(1998)11:3. PubMed: 9521420. 9521420

[48] Stenson PD, Mort M, Ball EV, Howells K, Phillips AD et al. (2009) The Human Gene Mutation Database: 2008 update. Genome Med 1: 13. doi:10.1186/gm13. PubMed: 19348700. 19348700 PMC2651586

[49] Ali H, Olatubosun A, Vihinen M (2012) Classification of mismatch repair gene missense variants with PON-MMR. Hum Mutat 33: 642-650. doi:10.1002/humu.22038. PubMed: 22290698. 22290698

[50] Terui H, Akagi K, Kawame H, Yura K (2013) CoDP: predicting the impact of unclassified genetic variants in MSH6 by the combination of different properties of the protein. J Biomed Sci 20: 25. doi:10.1186/1423-0127-20-25. PubMed: 23621914. 23621914 PMC3651391

[51] Pastrello C, Pin E, Marroni F, Bedin C, Fornasarig M et al. (2011) Integrated analysis of unclassified variants in mismatch repair genes. Genet Med 13: 115-124. doi:10.1097/GIM.0b013e3182011489. PubMed: 21239990. 21239990

[52] Arnold S, Buchanan DD, Barker M, Jaskowski L, Walsh MD et al. (2009) Classifying MLH1 and MSH2 variants using bioinformatic prediction, splicing assays, segregation, and tumor characteristics. Hum Mutat 30: 757-770. doi:10.1002/humu.20936. PubMed: 19267393. 19267393 PMC2707453

[53] Ward LD, Kellis M (2012) Interpreting noncoding genetic variation in complex traits and human disease. Nat Biotechnol 30: 1095-1106. doi:10.1038/nbt.2422. PubMed: 23138309. 23138309 PMC3703467

[54] Hitchins MP, Wong JJ, Suthers G, Suter CM, Martin DI et al. (2007) Inheritance of a cancer-associated MLH1 germ-line epimutation. N Engl J Med 356: 697-705. doi:10.1056/NEJMoa064522. PubMed: 17301300. 17301300

[55] Crépin M, Dieu MC, Lejeune S, Escande F, Boidin D et al. (2012) Evidence of constitutional MLH1 epimutation associated to transgenerational inheritance of cancer susceptibility. Hum Mutat 33: 180-188. doi:10.1002/humu.21617. PubMed: 21953887. 21953887

