# Supplementary figures and images for: Integrative Analysis of Hereditary Nonpolyposis Colorectal Cancer: the Contribution of Allele-Specific Expression and Other Assays to Diagnostic Algorithms
Source: PLoS One. 2013 Nov 20;8(11):e81194. doi: 10.1371/journal.pone.0081194 (PMC3835792; doi:10.1371/journal.pone.0081194)

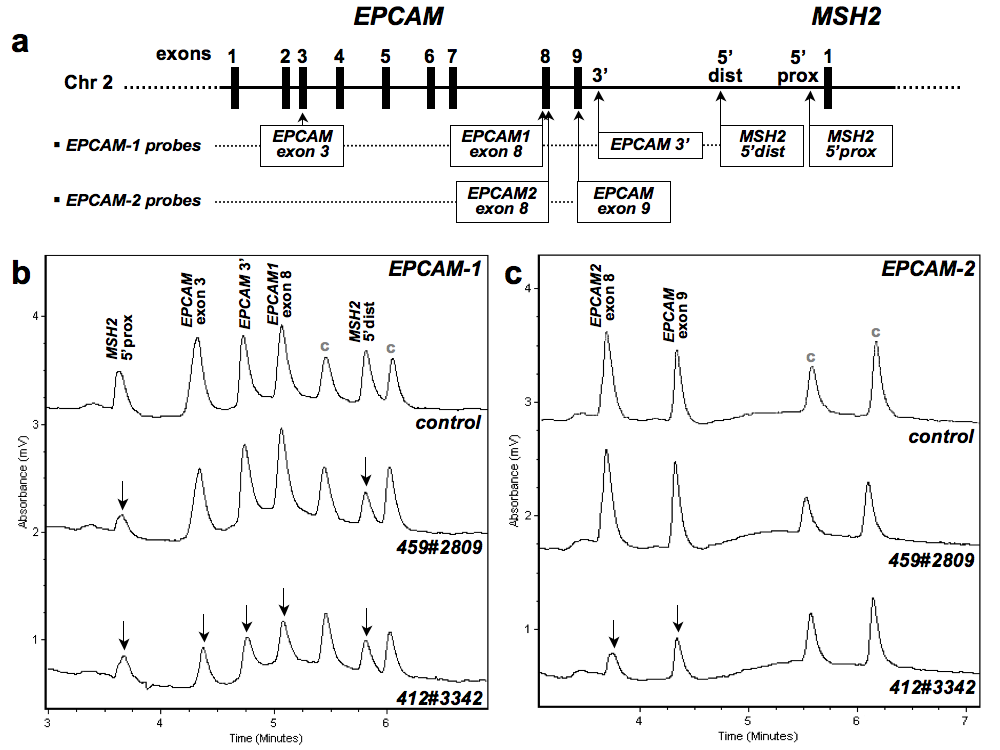

Supplement: Figure S1 — NFMP-HPLC assays for EPCAM genomic rearrangements. Probes for EPCAM-1 and EPCAM-2 assays are indicated based on their position in the region encompassing EPCAM and the 5’upstream region of MSH2 (panel a). Examples of EPCAM-1 (panel b) and EPCAM-2 (panel c) profiles are shown for control individuals (top of each panel) and 2 representative patients (459#2809, middle of each panel; 412#3342, bottom of each panel). Control peaks are labeled “c” and arrows indicate amplicons with decreased peak heights, indicative of genomic deletions. EPCAM-1 (panel b): in patient 459#2809 the chromatographic profile shows decreased peak heights for the 2 amplicons corresponding to the MSH2 5’ upstream region (proximal and distal); in patient 412#3342 all the EPCAM-MSH2 amplicons included in the assay show decreased peak heights compared to the control peaks. EPCAM-2 (panel c): in patient 459#2809 no alterations in the chromatographic profile are observed, confirming the absence of deletions in the EPCAM amplicons tested; in patient 412#33428 the peaks corresponding to the EPCAM probes show decreased heights compared to the control peaks, confirming the deletion of the EPCAM amplicons tested. (TIF) [file pone.0081194.s001.tif]

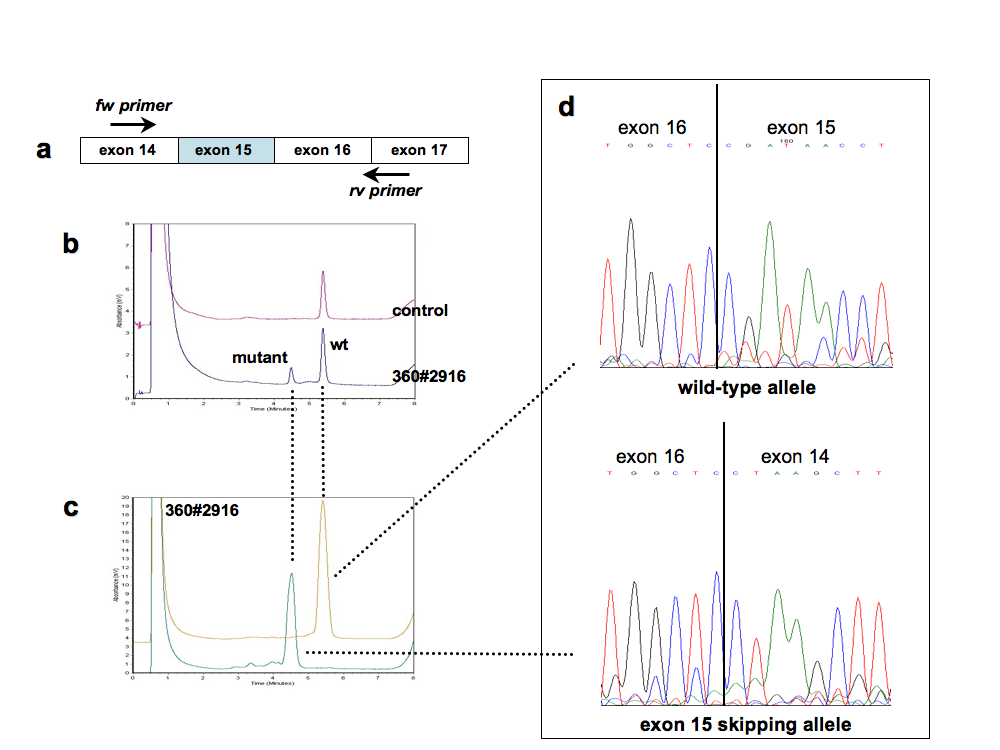

Supplement: Figure S2 — Molecular analysis of exon skipping in patient 360#2916. Panel a: Location of RT-PCR primers in MLH1 exons 14 and 17. Panel b: DHPLC chomatographic profiles obtained with cDNAs from patient 360#2916 and a control. The chromatogram of patient 360#2916 shows a major peak corresponding to the wt transcript and a minor peak corresponding to a less expressed shorter transcript (average allelic ratio 4.50, derived from 3 independent experiments). Panel c: DHPLC profiles derived from PCR amplification of the chromatographic fractions corresponding to the purified wt or shorter transcript. Panel d: Sequences corresponding to the two purified peaks (sequences of reverse strands are shown). The longer peak displays the wildtype sequence (top sequence), whereas the shorter peak shows the skipping of exon 15 (bottom sequence). (TIF) [file pone.0081194.s002.tif]
