# Supplementary material for: Integrative Analysis of Hereditary Nonpolyposis Colorectal Cancer: the Contribution of Allele-Specific Expression and Other Assays to Diagnostic Algorithms
Source: PLoS One. 2013 Nov 20;8(11):e81194. doi: 10.1371/journal.pone.0081194 (PMC3835792; doi:10.1371/journal.pone.0081194)
Supplement: Table S1 — Primers for NFMP-HPLC analysis of EPCAM-MSH2 rearrangements. The first NFMP-HPLC multiplex for EPCAM (EPCAM-1) consisted of 7 amplicons, including 2 located in exons 3 and 8 of EPCAM, 3 located within the intergenic MSH2-EPCAM region and 2 reference amplicons corresponding to MSH2 exon 9 and MLH1 exon 5. The second NFMP-HPLC multiplex for EPCAM (EPCAM-2) consisted of 4 amplicons, including 2 located in exons 8 and 9 of EPCAM and 2 reference amplicons corresponding to MSH2 exon 9 and MLH1 exon 5. The third NFMP-HPLC multiplex for EPCAM (EPCAM-3) differed from EPCAM-2 assay only for the reference amplicon that was located within a copy number invariant region in chromosome 2q36.1. Multiplex PCRs were performed using a touchdown protocol in a total of 23 cycles. Reactions were carried out on a GeneAmp PCR System 2720 thermocycler (Applied Biosystems), in a final volume of 10 µl containing 30-50 ng of template DNA and 0.5 unit of AmpliTaq Gold DNA polymerase (Applied Biosystems). (DOC) [file pone.0081194.s003.doc]

**Table S1. Primers for NFMP-HPLC analysis of *EPCAM-MSH2* rearrangements**

The first NFMP-HPLC multiplex for *EPCAM* (EPCAM-1) consisted of 7 amplicons, including 2 located in exons 3 and 8 of *EPCAM*, 3 located within the intergenic *MSH2*-*EPCAM* region and 2 reference amplicons corresponding to *MSH2* exon 9 and *MLH1* exon 5. The second NFMP-HPLC multiplex for *EPCAM* (EPCAM-2) consisted of 4 amplicons, including 2 located in exons 8 and 9 of *EPCAM* and 2 reference amplicons corresponding to *MSH2* exon 9 and *MLH1* exon 5. The third NFMP-HPLC multiplex for *EPCAM* (EPCAM-3) differed from EPCAM-2 assay only for the reference amplicon that was located within a copy number invariant region in chromosome 2q36.1. Multiplex PCRs were performed using a touchdown protocol in a total of 23 cycles. Reactions were carried out on a GeneAmp PCR System 2720 thermocycler (Applied Biosystems), in a final volume of 10 μl containing 30-50 ng of template DNA and 0.5 unit of AmpliTaq Gold DNA polymerase (Applied Biosystems).

| **Multiplex PCR** | **Genomic regions amplified (amplicon size, bp)** | **Multiplex PCR primers (5’>3’)** |
| --- | --- | --- |
| *EPCAM-1* | *EPCAM* – 5’ proximal *MSH2 -* intergenic region (86) | **f**: AAGGAGCTCTACTAAGGATGCGCGTCT |
|  |  | **r**: CGCACGCTGGTGACTTTAGCTACTG |
|  | *EPCAM* - exon 3 (116) | **f**: AGTGCAACGGCACCTCCATGTG |
|  |  | **r**: AGGCAGCCCCACTCACTAGGTT |
|  | *3’ EPCAM* - *MSH2 -* intergenic region (138) | **f**: TTGGCCTCCCTTGGCTGCTGATT |
|  |  | **r**: CGTTGGGAAAGGATTGAGAGTGGGG |
|  | *EPCAM* - exon 8 (161) | **f**: GAATGGCAAAGTATGAGAAGGCTGAGG |
|  |  | **r**: TGTCCCTAAGACCCTAAAGACAACAGT |
|  | *MLH1* - exon 5 - reference amplicon (187)^a^ | **f**: GATTTTCTCTTTTCCCCTTGGG |
|  |  | **r**: ACAAAGCTTCAACAATTTACTCT |
|  | *EPCAM* - 5’ distal *MSH2 -* intergenic region (209) | **f**: TATCAGAAATGGTAGTAGCTTCTCTAAAGG |
|  |  | **r**: CCTGGCTAATTTTTGTAATTTTTGTGGAG |
|  | *MSH2* - exon 9 - reference amplicon (229)^b^ | **f**: GGTGACTTGGAAAAGAAGATGC |
|  |  | **r**: GGGCTTGTTTAAATGACATC |
| *EPCAM-2* | *EPCAM* - exon 8 (83) | **f**: CAATAGTTGTCTTTCTTCCACTCAGG |
|  |  | **r**: GTAATCCATTTACCTCAGCCTTCTCA |
|  | *EPCAM* - exon 9 (110) | **f**: GTTTCAGATAAAGGAGATGGGTGA |
|  |  | **r**: CACATTTGTAATTTGTGTCCATTTGC |
|  | *MLH1* (187)^a^ and *MSH2* (229)^b^ - reference amplicons | *as in EPCAM-1* |
| *EPCAM-3* | *EPCAM* - exon 8 (83) and *EPCAM* - exon 9 (110) | *as in EPCAM-2* |
|  | *CN2-2 -* 2q36.1 - reference amplicon (154)^c^ | **f:** CTTAGGTTCCCACGGTTTGA |
|  |  | **r:** GCACTTGAAAGGTGCCTAGC |

f (forward); r (reverse)

^a^Primers for this reference amplicon derive from Charbonnier et al. [Charbonnier F, Raux G, Wang Q, Drouot N, Cordier F, et al. (2000) Detection of exon deletions and duplications of the mismatch repair genes in hereditary nonpolyposis colorectal cancer families using multiplex polymerase chain reaction of short fluorescent fragments. Cancer Res 60:2760-2763].

^b^Primers for this reference amplicon derive from Charbonnier et al. [Charbonnier F, Olschwang S, Wang Q, Boisson C, Martin C, et al. (2002) MSH2 in contrast to MLH1 and MSH6 is frequently inactivated by exonic and promoter rearrangements in hereditary nonpolyposis colorectal cancer. Cancer Res 62:848-853].

^c^Primers for this reference amplicon derive from Jeon et al. [Jeon JP, Shim SM, Nam HY, Ryu GM, Hong EJ, et al. (2010) Copy number variation at leptin receptor gene locus associated with metabolic traits and the risk of type 2 diabetes mellitus. BMC Genomics 1:426].
