# Supplementary material for: Integrative Analysis of Hereditary Nonpolyposis Colorectal Cancer: the Contribution of Allele-Specific Expression and Other Assays to Diagnostic Algorithms
Source: PLoS One. 2013 Nov 20;8(11):e81194. doi: 10.1371/journal.pone.0081194 (PMC3835792; doi:10.1371/journal.pone.0081194)
Supplement: Table S2 — Primers for analysis of germline MLH1 promoter methylation. (DOC) [file pone.0081194.s004.doc]

**Table S2. Primers for analysis of germline *MLH1* promoter methylation**

| **Primers for methylation-specific PCR (MSP)** | **Sequence (5’>3’)** |
| --- | --- |
| Forward - MLH1-MSP | ATTGGTTGGATATTTCGTATTTTTC^a^ |
| Reverse - MLH1-DEG-3 | CCTTCAACCAATCACCTCAATACC^a^ |
|  |  |
| **Primers for control PCR (unmethylated DNA)** | **Sequence (5’>3’)** |
| Forward - MLH1-F | ATTGGTTGGATATTTTGTATTTTTT |
| Reverse - MLH1-DEG-3 | *as above* |

^a^Kindly provided by Suter et al. [30]
