# Supplementary material for: Integrative Analysis of Hereditary Nonpolyposis Colorectal Cancer: the Contribution of Allele-Specific Expression and Other Assays to Diagnostic Algorithms
Source: PLoS One. 2013 Nov 20;8(11):e81194. doi: 10.1371/journal.pone.0081194 (PMC3835792; doi:10.1371/journal.pone.0081194)
Supplement: Table S3 — Comparison between results of ASE analyses performed using 32P-labeled primers or DHPLC. (DOC) [file pone.0081194.s005.doc]

**Table S3. Comparison between results of ASE analyses performed using ^32^P-labeled primers or DHPLC**

| Patients | Sequence variant |  | Peak ratios | Normalized allelic ratios |
| --- | --- | --- | --- | --- |
|  |  |  | mean (SE) | mean (SE) |
| 360#2916 | *MLH1* c.655A>G | ***^32^P*** | 4.32 (0.30) | 3.71 (0.28) |
|  |  | ***DHPLC^a^*** | 4.74 (0.34) | 4.93 (0.35) |
| 360#2916 | *MLH1* c.1852-3delAAinsGC | ***^32^P*** | 4.84 (0.10) | 4.57 (0.10) |
|  |  | ***DHPLC^a^*** | 5.50 (0.27) | 5.62 (0.27) |
| 96#1636 | *MLH1* c.655A>G | ***^32^P*** | 0.78 (0.09) | 0.90 (0.09) |
|  |  | ***DHPLC*** | 0.95 (0.05) | 1.17 (0.06) |

^a^Previously reported in Aceto et al. [23]
