# Supplementary material for: Integrative Analysis of Hereditary Nonpolyposis Colorectal Cancer: the Contribution of Allele-Specific Expression and Other Assays to Diagnostic Algorithms
Source: PLoS One. 2013 Nov 20;8(11):e81194. doi: 10.1371/journal.pone.0081194 (PMC3835792; doi:10.1371/journal.pone.0081194)
Supplement: Table S4 — Primers for ASE assays developed in this study. (DOC) [file pone.0081194.s006.doc]

# Table S4. Primers for ASE assays developed in this study^a^

| Gene | **Variant** | **gDNA-PCR primers (5’>3’)** | **cDNA-PCR primers (5’>3’)** | | **PE primers (5’>3’)** | **ddNTP** |
| --- | --- | --- | --- | --- | --- | --- |
| ***MSH2*** | **c.278delTT** | **f:** CTTGAACATGTAATATCTCAAATCTG | **1st round** | **f:** GCCGCCCGGGAGGTGTTCAAG | **f:** CAACTCTATACTGACGAACC | **ddTTP** |
|  |  |  |  | **r:** CTTGGATGCCTTATTTCCAG |  |  |
|  |  | **r:** TTATTTTTCTACTCTTAAAAAAATAAC | **2nd round** | **f:** ACCCAGGGGGTGATCAAGTACA |  |  |
|  |  |  |  | **r:** CTTATAAACTTCAACTCTATACTGACG |  |  |
|  | **c.984C>T** | **f:** GGTTCTGTTGAAGATACCACTG | **1st round** | **f:** TAGGGTTCTGTTGAAGATACCA | **f:** CACTGGCTCTCAGTCTCTGGCT | **ddTTP** |
|  |  |  |  | **r:** CAAAAGCTTCCACTAAATTCAAT |  |  |
|  |  | **r:** CTGTTCTTATCCATGAGAGGC | **2nd round** | **f:** GGTTCTGTTGAAGATACCACTG |  |  |
|  |  |  |  | **r:** TTCCACTAAATTCAATCTCTCC |  |  |
| ***MSH6*** | **c.540T>C** | **f:** CGTGAGCCTCTGCACCCGG^b^ | **1st round** | **f:** GCCTTGTCTGGTTTACAAC^b^ | **f:** ATACTGAGAGCAATGCAACGTGC | **ddTTP** |
|  |  |  |  | **r:** CTTGTGTCTTAGGCTGTAC^b^ |  |  |
|  |  | **r:** CCCATCACCCTAACATAAA^b^ | **2nd round** | **f:** CTTGTCTGGTTTACAACCA |  |  |
|  |  |  |  | **r:** TGTCTTAGGCTGTACTTCC |  |  |

f (forward); r (reverse).

^a^Templates for primer extension were generated either by single step or nested PCR protocols as described [25]. For cDNA amplification, primers employed for 1^st^ round PCR lie in contiguous exons to avoid amplification of contaminating gDNA that might be present despite DNAse treatment.

^b^These primers derive from Renkonen et al. [26]
