# Supplementary material for: Integrative Analysis of Hereditary Nonpolyposis Colorectal Cancer: the Contribution of Allele-Specific Expression and Other Assays to Diagnostic Algorithms
Source: PLoS One. 2013 Nov 20;8(11):e81194. doi: 10.1371/journal.pone.0081194 (PMC3835792; doi:10.1371/journal.pone.0081194)
Supplement: Table S5 — MSH2, MLH1 and MSH6 nucleotide variants detected. (DOC) [file pone.0081194.s007.doc]

**Table S5. *MSH2, MLH1* and *MSH6* nucleotide variants detected**

| **Variant type** | **Patients** | MSI | IHC | Gene | **DNA change and consequence^a^** |
| --- | --- | --- | --- | --- | --- |
|  |  |  |  |  |  |
| ***Nonsense*** |  |  |  |  |  |
|  | 705#3035 | na | MLH1 | MLH1 | **c.1367C>A (p.Ser456*)** |
|  | 711#2495 | MSI-H | MLH1 | MLH1 | c.1459C>T (p.Arg487*) |
|  | TO9726 | MSI-H | na | MLH1 | c.2040C>A (p.Cys680*) |
|  | TO0225 | na | na | MLH1 | c.2040C>A (p.Cys680*) |
|  | 350#1933 | MSI-H | MLH1 | MLH1 | c.676C>T (p.Arg226*) |
|  | 1251#3260 | MSI-H | MSH2 | MSH2 | **c.374C>T (p.Gln125*)** |
|  | 1420#3343 | na | MSH2 | MSH2 | c.868G>T (p.Glu290*) |
|  | 1205#BA | MSI-H | MLH1 | MSH2 | c.1215C>A (p.Tyr405*) |
|  | GE0330 | na | na | MSH2 | c.1216C>T (p.Arg406*) |
|  | 1515#3442 | MSI-H | MSH2 | MSH2 | c.1255C>T (p.Gln419*) |
|  | 1008#2829 | na | MSH2 | MSH2 | **c.1757C>G (p.Ser586*)** |
|  | LCH-19 | MSI-H | MSH2 | MSH2 | c.2245G>T (p.Glu749*) |
|  | GE9726 | MSI-H | na | MSH2 | c.2536C>T (p.Gln846*) |
|  | 601#2307 | na | na | MSH6 | c.3013C>T (p.Arg1005*) |
| ***Frameshift*** |  |  |  |  |  |
|  | GDLG-31#III-11^b^ | na | na | MLH1 | c.954delC (p.His318Glnfs*49) |
|  | 602#2416 | na | na | MLH1 | c.1011delC (p.Asp338Ilefs*29) |
|  | GE9911 | na | na | MLH1 | c.1011delC (p.Asp338Ilefs*29) |
|  | 1068#3015 | MSI-H | MLH1 | MLH1 | c.1050delA (p.Gly351Aspfs*16) |
|  | 359#2578 | na | na | MLH1 | **c.1639_1643dupTTATA (p.Leu549Tyrfs*44)** |
|  | LCH-59 | MSI-H | MSH2 | MLH1 | **c.1679delT (p.Phe560Serfs*31)** |
|  | TO0012 | na | na | MLH1 | **c.1888_1892delATTGA (p.Ile630*)** |
|  | 19#719 | MSI-H | MSH2 | MSH2 | c.1444dupA (p.Arg482Lysfs*6) |
|  | GDLM-9#II-2^c^ | MSI-H | MSH2 | MSH2 | **c.1549_1550delGCinsT (p.Ala517Tyrfs*9)** |
|  | 334#1170 | MSI-H | MSH2 | MSH2 | c.278_279delTT (p.Leu93Profs*6) |
|  | 357#2038^d^ | MSI-H | na | MSH2 | c.2294delC (p.Ala765Valfs*47) |
|  | 1074#3001 | na | na | MSH2 | c.1059delG (p.Asn354Thrfs*3) |
|  | GE9804 | MSI-H | na | MSH2 | c.1705_1706delGA (p.Glu569Ilefs*2) |
|  | 1157#834 | na | na | MSH2 | c.119delG (p.Gly40Alafs*24) |
|  | 1200#3221 | na | na | MSH6 | **c.738_741delAAAA (p.Lys246Asnfs*32)** |
| ***Splice site*** |  |  |  |  |  |
|  | 1077#2979 | na | MLH1 | MLH1 | **c.453+1G>A** |
|  | 668#2371^d^ | MSI-H | MLH1 | MLH1 | c.545+3A>G^e^ |
|  | F102 | MSI-H | na | MLH1 | c.546-2A>G^f^ |
|  | GE9914 | MSI-H | na | MLH1 | c.677+1G>A |
|  | 986#3487 | MSI-H | MLH1 | MLH1 | **c.1731+4A>G**^g^ |
|  | 360#2916^h^ | MSI-H | MLH1/MSH6 | MLH1 | **c.1731+4A>G**^g^ |
|  | 162#2696 | na | na | MLH1 | c.1559-2A>G^i^ |
|  | LCH-88 | MSI-H | na | MLH1 | c.1731G>A (p.Ser577Ser)^j^ |
|  | 324#R10 | na | na | MLH1 | c.1896G>A (p.Glu632Glu)^k^ |
|  | LCH-57 | MSI-H | MLH1 | MLH1 | c.1989G>T (p.Glu663Asp)^l^ |
|  | 670#2413 | MSI-H | MLH1 | MLH1 | c.1989G>T (p.Glu663Asp)^l^ |
|  | 737#2838 | MSI-H | na | MLH1 | c.1989G>T (p.Glu663Asp)^l^ |
|  | 337#2224 | na | na | MLH1 | c.1989G>T (p.Glu663Asp)^l^ |
|  | LCH-86 | na | na | MSH2 | c.212-1G>A |
|  | 298#668/1584 | MSI-H | MSH2 | MSH2 | c.1077-2A>C |
|  | 727#AA | MSI-H | MSH2 | MSH2 | **c.942+2T>A** |
|  | GDLM-2#III-1^b^ | MSI-H | MSH2 | MSH2 | c.942+3A>T^m^ |
|  | 349#1581^d^ | MSI-H | MSH2 | MSH2 | c.942+3A>T^m^ |
|  | 667#2412^d^ | MSI-H | MSH2 | MSH2 | c.942+3A>T^m^ |
|  | SI9744 | MSI-H | na | MSH2 | c.942+3A>T^m^ |
|  | GE0410 | na | na | MSH2 | c.942+3A>T^m^ |
|  | LCH-58 | MSI-H | MSH2 | MSH2 | c.2005+3_2005+14del12^n^ |
| ***In-frame deletion*** |  |  |  |  |  |
|  | GDLG-29#III-8^b,o^ | na | na | MLH1 | c.1852_1854delAAG (p.Lys618del)^p^ |
|  | 297#875 | MSI-H | MLH1 | MLH1 | c.1852_1854delAAG (p.Lys618del)^p^ |
|  | 1459#3324 | MSI-H | MLH1 | MLH1 | c.1852_1854delAAG (p.Lys618del)^p^ |
|  | 463#3031 | na | na | MLH1 | c.1852_1854delAAG (p.Lys618del)^p^ |
|  | 1138#3149 | MSI-H | na | MLH1 | c.1852_1854delAAG (p.Lys618del)^p^ |
|  | 1301#3323 | MSI-H | MSH2 | MSH2 | **c.2519_2530del12 (p.Val840_Cys843del)** |
|  | 1256#3479 | MSI-H | MSH2 | MSH2 | c.1786_1788delAAT (p.Asn596del)^q^ |
| ***Missense*** |  |  |  |  |  |
|  | 307#2619 | MSI-H | MLH1 | MLH1 | c.199G>A (p.Gly67Arg)^p,r,s^ |
|  | LCH-1 | MSI-H | MLH1 | MLH1 | c.301G>A (p.Gly101Ser)^s^ |
|  | 338#1489^d^ | MSI-H | MLH1/MSH6 | MLH1 | c.382G>C (p.Ala128Pro)^r,s^ |
|  | 311#2042^d^ | MSI-H | ni | MLH1 | c.731G>A (p.Gly244Asp)^r,s,t^ |
|  | LES1#LP | MSI-H | na | MLH1 | c.731G>A (p.Gly244Asp)^r,s,t^ |
|  | SI9606 | MSI-H | na | MLH1 | c.911A>T (p.Asp304Val)^r,s^ |
|  | LCH-15 | na | ni | MLH1 | c.1918C>T (p.Pro640Ser)^u^ |
|  | 814#DGR | MSI-H | na | MSH2 | **c.376G>A (p.Gly126Ser)**^v^ |
|  | GDLG-49#IV-2^b^ | MSI-H | MSH2 | MSH2 | c.1024G>A (p.Val342Ile)^u^ |
|  | LCH-93 | MSI-H | na | MSH2 | c.1046C>G (p.Pro349Arg)^s^ |
|  | 1206#GE | MSI-H | MSH2 | MSH2 | c.1046C>G (p.Pro349Arg)^s^ |
|  | 319#1004 | MSI-H | MSH2 | MSH2 | c.1046C>G (p.Pro349Arg)^s^ |
|  | 1080#2974 | MSI-H | na | MSH2 | c.2089T>C (p.Cys697Arg)^s,t,w^ |
|  | 814#DGR | MSI-H | na | MSH2 | **c.2251G>C (p.Gly751Arg)**^x^ |
|  | 1070#2957 | MSI-H | MSH2 | MSH2 | **c.2287G>C (p.Ala763Pro)**^v^ |
|  |  |  |  |  |  |

na, not available; ni, IHC not informative.

NOTE. The following variants detected in the present study were considered nonpathogenic based on frequency in CRC-unaffected individuals [21], *in silico* and/or functional assays ([www.mmruv.info](http://www.mmruv.info/)): the c.965G>A (rs4987188) and the c.984C>T (rs4987189) variants of *MSH2*; the c.655G>A (rs1799977), the c.1852_1853delAAinsGC, the c.2306_2308delCTT, the c.1090A>G (rs63749864) and the c.702G>A (rs35908749) variants of *MLH1*; the c.540T>C (rs1800935) and the c.276A>G (rs1800932) variants of *MSH6*.

^a^Novel variants are in bold.

^b^Previously reported in Curia et al. [23]

^c^Previously reported in Curia et al. [23] as negative for MMR gene variants, but showing imbalanced germline ASE of *MSH2*.

^d^Previously reported in Pensotti et al. [33]

^e^Nucleotide variant previously verified to associate with skipping of *MLH1* exon 6 [33].

^f^Nucleotide variant previously verified to associate with skipping of *MLH1* exons 6 and 7 [34].

^g^Nucleotide variant predicted to generate decreased donor site strength by HSF and FruitFly, but considered a “polymorphisms” or of “unknown pathogenicity” by Mutation taster and Alamut, respectively. It was verified to associate with altered ASE and skipping of *MLH1* exon 15 in this study.

^h^Previously reported in Aceto et al. [25] as negative for MMR gene variants, but showing imbalanced germline ASE of *MLH1*.

^i^Nucleotide variant previously verified to associate with skipping of *MLH1* exons 14 and 15 [35].

^j^Nucleotide variant previously verified to associate with skipping of *MLH1* exon 15 [36].

^k^Nucleotide variant previously verified to associate with skipping of *MLH1* exon 16 [37].

^l^Nucleotide variant previously verified to associate with skipping of *MLH1* exon 17 [36].

^m^Nucleotide variant previously verified to associate with skipping of *MSH2* exon 5 [38].

^n^Previously reported as pathogenic, but evidence of pathogenicity not discussed (see InSiGHT database, <http://www.insight-group.org/variants/database/>, Sanchez de Abajo et al., 2006). Predicted to affect splicing by HSF, FruitFly, Mutation Taster and Alamut software tools (this study).

^o^Previously reported in Aceto et al. [25]

^p^Classified as pathogenic using a multifactorial likelihood model [44].

^q^Previously reported to affect protein function [39].

^r^Previously reported to affect protein function [41].

^s^Previously classified as deleterious based on MAPP-MMR [42] and PON-MMR *in silico* analyses.

^t^Classified as pathogenic based on a qualitative classifier [43].

^u^Previously classified as deleterious based on MAPP-MMR [42], but considered as unclassified variant by PON-MMR.

^v^Predicted to be deleterious on protein function using SIFT, PolyPhen-2, Mutation Taster, Alamut, MAPP-MMR and PON-MMR software tools (this study).

^w^Previously reported to affect protein function [40].

^x^Predicted to be deleterious on protein function using SIFT, PolyPhen-2, Mutation Taster, Alamut, MAPP-MMR and PON-MMR software tools (this study). This nucleotide variant is novel, but is predicted to induce the same aminoacid change of a previously described variant c.2251G>A (p.Gly751Arg) that was classified as deleterious [40,42].
