# Supplementary material for: Integrative Analysis of Hereditary Nonpolyposis Colorectal Cancer: the Contribution of Allele-Specific Expression and Other Assays to Diagnostic Algorithms
Source: PLoS One. 2013 Nov 20;8(11):e81194. doi: 10.1371/journal.pone.0081194 (PMC3835792; doi:10.1371/journal.pone.0081194)
Supplement: Table S6 — Analysis of genomic rearrangements. (DOC) [file pone.0081194.s008.doc]

Table S6. Analysis of genomic rearrangements

|  |  |  |  |  |  |
| --- | --- | --- | --- | --- | --- |
| **Patients** | **MSI** | **MLPA^a^** | **NFMP-HPLC** | **Other methods** | **Breakpoints coordinates^b^** |
|  |  |  |  |  |  |
|  |  |  |  |  |  |
| 600#2237^c^ | MSI-H | *MSH2* Del exons 4-6 | *MSH2* Del exons 4-6 | PCR | c.645+967_1076+5075del10166 |
| 363#2541^c^ | MSI-H | *MSH2* Del exons 9-10 | *MSH2* Del exons 9-10 | PCR | c.1387-3976_1662-364del11547 |
| 903#2630^c^ | MSI-H | *MSH2* Del exon 3 | *MSH2* Del exon 3 | Long-range PCR | c.367-452_646-722del2051 |
| 985#2683^c,d^ | nd | *MLH1* Del exon 6 | *MLH1* Del exon 6 | LOC-CNV, PCR | c.454-432_546-1030del2409 |
| 459#2809 | MSI-H | *MSH2* Del exons 1-8^c^ | *MSH2* Del 5’upstream region - exon 8 | nd | nd |
| 83#3103^c^ | nd | *MLH1* Del exon 1 | *MLH1* Del exon 1 | RT-PCR^e^ | nd |
| 1218#3238^d^ | MSI-H | *MLH1* Dup exons 2-3 | *MLH1* Dup exons 2-3 | LOC-CNV | c.116+624_306+1086dup7853 |
|  |  |  |  | Long-range PCR |  |
| 1293#3286^d^ | MSI-H | *MLH1* Del exon 6 | *MLH1* Del exon 6 | LOC-CNV | c.454-665_545+52del809insA |
|  |  |  |  | Long-range PCR |  |
| 412#3342 | MSI-H | *MSH2* Del exons 1-7 | *EPCAM* Del exon 3 - *MSH2* exon 7 | nd | nd |
| 309#3478 | MSI-H | *MSH2* Del exons 9-10 | *MSH2* Del exons 9-10 | nd | nd |
| 476#R26 | MSI-H | *MSH2* Del exons 1-6 | *MSH2* Del exons 1-6 | nd | nd |
| LCH-23 | nd | *MSH2* Del exon 1 | *MSH2* Del exon 1 | nd | nd |
| GDLM-7#III-3^c^ | MSI-H | *MSH2* Del exon 7 | *MSH2* Del exon 7 | RT-PCR^e^ | c.1077-?_1276+?del |
| GDLG-18#III-19^c^ | MSI-H | *MLH1* Del exon 3 | *MLH1* Del exon 3 | PCR | c.208-1714_306+641del2454ins8 |
| GE0201 | nd | *MSH2* Del exon 16 | *MSH2* Del exon 16 | nd | nd |
|  |  |  |  |  |  |

nd, not done.

^a^MLPA kit used for these analyses did not include probes corresponding to *EPCAM*.

^b^Breakpoint coordinates are defined according to the gDNA sequences obtained from the Ensembl Genome Browser (www.ensembl.org) [*MSH2* (ENSG00000095002); *MLH1* (ENSG00000076242)]. Numbering is based on the cDNA sequences of *MSH2* (NM_000251.1) and *MLH1* (NM_000249.2), according to the approved systematic nomenclature that follows the +1 rule of the A from ATG translation initiation codon.

^c^Previously reported in De Lellis et al. [28]

^d^Previously reported in De Lellis et al. [29]

^e^cDNA sequencing indicated the loss of expression of one *MLH1* allele in patient 83#3103 and the deletion of exon 7 from transcripts in patient GDLM-7#III-3 [28].
