# Supplementary material for: Integrative Analysis of Hereditary Nonpolyposis Colorectal Cancer: the Contribution of Allele-Specific Expression and Other Assays to Diagnostic Algorithms
Source: PLoS One. 2013 Nov 20;8(11):e81194. doi: 10.1371/journal.pone.0081194 (PMC3835792; doi:10.1371/journal.pone.0081194)
Supplement: Table S7 — Correlations between the results of ASE and other analyses. (DOC) [file pone.0081194.s009.doc]

**Table S7. Correlations between the results of ASE and other analyses**

| **Patients** | **IHC** | **MSI** | **Pathogenic germline variant** | **ASE analysis** | | | | | |
| --- | --- | --- | --- | --- | --- | --- | --- | --- | --- |
|  |  |  |  | ***MLH1*** | | ***MSH2*** | | ***MSH6^a^*** | |
|  |  |  |  | **ASE** | **ASE marker** | **ASE** | **ASE marker** | **ASE** | **ASE marker** |
|  |  |  |  |  |  |  |  |  |  |
| GDLM-2#III-1 | MSH2 | MSI-H | *MSH2* c.942+3A>T | N | c.655A>G | ND |  | Ho |  |
| LCH-8 | MSH2 | MSI-H |  | N | c.655A>G | N | c.984C>T | Ho |  |
| GDLM-9#II-2 | MSH2 | MSI-H | *MSH2* c.1549_1550delGCinsT (p.Ala517Tyrfs*9) | Ho |  | U^b^ | c.965G>A | Ho |  |
| GDLV-11#II-9 | MLH1 | MSI-H |  | N^b^ | c.655A>G | Ho |  | Ho |  |
| GDLG-18#III-19 | ni | MSI-H | *MSH2* Del exon 3 | N | c.655A>G | ND |  | ND | c.540T>C |
| GDLG-20#II-1 | MLH1 | MSI-H |  | U^b^ | c.702G>A | Ho |  | Ho |  |
|  |  |  |  | U^b^ | c.2306_8delCTT | Ho |  | Ho |  |
| LCH-27 | MSH2 | MSI-H |  | U | c.655A>G | Ho |  | ND | c.276A>G |
| GDLG-29#III-8 | na | na | *MLH1* c.1852_1854delAAG (p.Lys618del) | N^c^ | c.1852_1854delAAG | Ho |  | ND | c.540T>C |
| GDLG-31#III-11 | na | na | *MLH1* c.954delC (p.His318Glnfs*49) | U | c.655A>G | Ho |  | Ho |  |
| GDLG-49#IV-2 | MSH2 | MSI-H | *MSH2* c.1024G>A (p.Val342Ile) | Ho |  | N^b^ | c.1024G>A | ND | c.540T>C |
| LCH-51 | na | na |  | N | c.655A>G | Ho |  | Ho |  |
| GDLV-52#II-2 | MLH1 | MSI-H |  | U^b^ | c.1090A>G | Ho |  | Ho |  |
| LCH-59 | MSH2 | MSI-H | *MLH1* c.1679delT (p.Phe560Serfs*31) | N | c.655A>G | Ho |  | Ho |  |
| GE9804 | na | MSI-H | *MSH2* c.1705_1706delGA (p.Glu569Ilefs*2) | N | c.655A>G | ND |  | Ho |  |
| GE9903 | na | MSI-H |  | Ho |  | N^c^ | c.965G>A | Ho |  |
| 360#2916 | MLH1/ | MSI-H | *MLH1* c.1731+4A>G | U^c^ | c.655A>G | Ho |  | Ho |  |
|  | MSH6 |  |  | U^c^ | c.1852_3delAAinsGC | Ho |  | Ho |  |
| 96#1636 | MLH1 | MSI-H |  | N | c.655A>G | Ho |  | Ho |  |
| 334#1170 | MSH2 | MSI-H | *MSH2* c.278_279delTT (p.Leu93Profs*6) | Ho |  | U | c.278_279delTT | Ho |  |
| 359#2578 | na | na | *MLH1* c.1639_1643dupTTATA (p.Leu549Tyrfs*44) | N | c.655A>G | Ho |  | Ho |  |
| 314#1200 | ni | na |  | N | c.655A>G | Ho |  | ND | c.540T>C |
| 1082#2982 | na | na |  | N | c.655A>G | Ho |  | N | c.540T>C |
| 83#3103 | na | na | *MLH1* Del exon 1 | U^d^ | c.655A>G | Ho |  | Ho |  |
|  |  |  |  |  |  |  |  |  |  |

na, not available; ni, IHC not informative; N, balanced or modest imbalance; U, imbalanced (ASE value <0.5 or > 2); Ho, homozygote for the marker used in the ASE analysis; ND, not done.

^a^ASE in *MSH6* could only be analyzed in patient 1082#2982, because for other informative patients RNA templates had been exhausted in previous *MLH1* and *MSH2* ASE analyses.

^b^Previously reported in Curia et al. [23]

^c^Previously reported in Aceto et al. [25]

^d^Previously reported as loss of one *MLH1* allele by sequencing [28] and confirmed in the present study by DHPLC-based primer extension.
